# Adaptation of the Risk Analysis Index for Frailty Assessment Using Diagnostic Codes

**DOI:** 10.1001/jamanetworkopen.2024.13166

**Published:** 2024-05-24

**Authors:** Alis J. Dicpinigaitis, Yekaterina Khamzina, Daniel E. Hall, Hasan Nassereldine, Jason Kennedy, Christopher W. Seymour, Meic Schmidt, Katherine M. Reitz, Christian A. Bowers

**Affiliations:** 1 Department of Neurology, New York Presbyterian–Weill Cornell Medical Center, New York, New York; 2 Bowers Neurosurgical Frailty and Outcomes Data Science Lab, Albuquerque, New Mexico; 3 Department of Surgery, University of Pittsburgh, Pittsburgh, Pennsylvania; 4 Department of Surgery, Veterans Affairs Pittsburgh Healthcare System, Pittsburgh, Pennsylvania; 5 Center for Health Equity Research and Promotion, Veterans Affairs Pittsburgh Healthcare System, Pittsburgh, Pennsylvania; 6 Wolff Center, UPMC, Pittsburgh, Pennsylvania; 7 Clinical Research, Investigation, and Systems Modeling of Acute Illness (CRISMA) Center, Pittsburgh, Pennsylvania; 8 Department of Critical Care Medicine, University of Pittsburgh, Pittsburgh, Pennsylvania; 9 Division of Vascular Surgery, University of Pittsburgh, Pittsburgh, Pennsylvania

## Abstract

**Importance:**

Frailty is associated with adverse outcomes after even minor physiologic stressors. The validated Risk Analysis Index (RAI) quantifies frailty; however, existing methods limit application to in-person interview (clinical RAI) and quality improvement datasets (administrative RAI).

**Objective:**

To expand the utility of the RAI utility to available *International Classification of Diseases, Tenth Revision, Clinical Modification* (*ICD-10-CM*) administrative data, using the National Inpatient Sample (NIS).

**Design, Setting, and Participants:**

RAI parameters were systematically adapted to *ICD-10-CM* codes (RAI-ICD) and were derived (NIS 2019) and validated (NIS 2020)*.* The primary analysis included survey-weighed discharge data among adults undergoing major surgical procedures. Additional external validation occurred by including all operative and nonoperative hospitalizations in the NIS (2020) and in a multihospital health care system (UPMC, 2021-2022). Data analysis was conducted from January to May 2023.

**Exposures:**

RAI parameters and in-hospital mortality.

**Main Outcomes and Measures:**

The association of RAI parameters with in-hospital mortality was calculated and weighted using logistic regression, generating an integerized RAI-ICD score. After initial validation, thresholds defining categories of frailty were selected by a full complement of test statistics. Rates of elective admission, length of stay, hospital charges, and in-hospital mortality were compared across frailty categories. C statistics estimated model discrimination.

**Results:**

RAI-ICD parameters were weighted in the 9 548 206 patients who were hospitalized (mean [SE] age, 55.4 (0.1) years; 3 742 330 male [weighted percentage, 39.2%] and 5 804 431 female [weighted percentage, 60.8%]), modeling in-hospital mortality (2.1%; 95% CI, 2.1%-2.2%) with excellent derivation discrimination (C statistic, 0.810; 95% CI, 0.808-0.813). The 11 RAI-ICD parameters were adapted to 323 *ICD-10-CM* codes. The operative validation population of 8 113 950 patients (mean [SE] age, 54.4 (0.1) years; 3 148 273 male [weighted percentage, 38.8%] and 4 965 737 female [weighted percentage, 61.2%]; in-hospital mortality, 2.5% [95% CI, 2.4%-2.5%]) mirrored the derivation population. In validation, the weighted and integerized RAI-ICD yielded good to excellent discrimination in the NIS operative sample (C statistic, 0.784; 95% CI, 0.782-0.786), NIS operative and nonoperative sample (C statistic, 0.778; 95% CI, 0.777-0.779), and the UPMC operative and nonoperative sample (C statistic, 0.860; 95% CI, 0.857-0.862). Thresholds defining robust (RAI-ICD <27), normal (RAI-ICD, 27-35), frail (RAI-ICD, 36-45), and very frail (RAI-ICD >45) strata of frailty maximized precision (F1 = 0.33) and sensitivity and specificity (Matthews correlation coefficient = 0.26). Adverse outcomes increased with increasing frailty.

**Conclusion and Relevance:**

In this cohort study of hospitalized adults, the RAI-ICD was rigorously adapted, derived, and validated. These findings suggest that the RAI-ICD can extend the quantification of frailty to inpatient adult *ICD-10-CM*–coded patient care datasets.

## Key Points

**Question:**

Can the Risk Analysis Index (RAI), a validated frailty assessment, be adapted to and validated in inpatient administrative data ubiquitously available in retrospective datasets and electronic health records?

**Findings:**

In this cohort study that included data from more than 9.5 million hospitalized adults, when the RAI parameters were adapted to the *International Statistical Classification of Diseases, Tenth Revision, Clinical Modification *(RAI-ICD), increasing RAI-ICD scores were associated with an increase in hospital length of stay, hospital charges, and in-hospital mortality.

**Meaning:**

These findings suggest that the rigorously adapted, derived, and validated RAI-ICD extends the quantification of frailty to administrative inpatient hospitalization data.

## Teaser

This cohort study examines the utility of adapting the Risk Analysis Index for Frailty using diagnostic codes and whether the adapted version is associated with adverse outcomes.

## Introduction

Frailty is a syndrome defined by vulnerability to stressors, resulting in adverse outcomes accounting for an increased risk of health care utilization, loss of functional independence, and mortality.^1,2,3,4,5,6,7^ Multiple validated instruments quantify frailty using varying statistical and conceptual models, each applicable to discrete and disparate data sources.^2,8^ Applying different frailty models leads to inconsistent results and variable conclusions with an inability to equitably compare findings across studies, limiting overall generalizability. Therefore, a reliable conceptual framework and measure of frailty applicable to numerous clinical settings and datasets is needed.

The Risk Analysis Index (RAI) represents a robust metric based on the deficit accumulation frailty model, reliably projecting short-term and long-term mortality in both surgical and nonsurgical adult populations.^9^ Initially developed and prospectively validated as a 14-item questionnaire, the RAI is the only frailty assessment proven feasible for real-time, point-of-care frailty assessment of predominantly robust populations. When used to inform preoperative decision-making, the clinical RAI (RAI-C) is associated with the pragmatic improvement of postoperative outcomes, including reduced mortality.^10,11^ The prospective screening RAI-C is complemented by the administrative RAI (RAI-A), adapted for retrospective use of available frailty-associated variables exclusively applicable to surgical quality datasets.^12,13,14^ To date, the RAI has not been applied to, or optimized for use with, other administrative data based on the globally available *International Classification of Diseases, Tenth Revision, Clinical Modification* (*ICD-10-CM*) codes. We aim to map the conceptual framework of the validated RAI parameters to data ubiquitously available in inpatient administrative data, including *ICD-10-CM* codes, thereby allowing frailty investigation to substantially expand beyond in-person evaluation and surgical quality datasets to a broader assessment of surgical and nonsurgical diagnoses.

## Methods

This cohort study followed the Standards for Reporting of Diagnostic Accuracy (STARD) reporting guideline^15^ and Strengthening the Reporting of Observational Studies in Epidemiology (STROBE) reporting guideline. In 4 steps, we adapted, derived, stratified, and validated the RAI using *ICD-10-CM* codes (RAI-ICD) in the National Inpatient Sample (NIS). Secondary external validation occurred using data abstracted from 14 community and academic hospitals in an integrated health care system (UPMC). The secondary analysis of both datasets was reviewed by the University of Pittsburgh Human Research Protection Office with usage exempt from human participants review (NIS) or with a waiver of informed consent (UPMC) because the study used deidentified data in accordance with the Common Rule. All data were presented in accordance with the NIS, Healthcare Cost and Utilization Project (HCUP), and Agency for Healthcare Research and Quality use agreements.^16^

### Data Sources

The primary analysis was completed using the NIS (2019-2020). The NIS, developed and maintained by HCUP, includes unweighted data for approximately 7 000 000 annual hospitalizations in the US regardless of expected payer, reflecting a 20% stratified sample of HCUP-participating, nonfederal, acute-care hospitals.^16^ Data elements include patient demographic and hospital characteristics; 40 *ICD-10-CM* and 20 *International Statistical Classification of Diseases, Tenth Revision, Procedure Coding System (ICD-10-PCS)* codes; and discharge disposition. Race and ethnicity are categorized as 1 data element. Race and ethnicity were categorized as Asian, Black, White, other (defined as Hispanic, Native American, or any other race or ethnicity not otherwise specified), and missing. Race and ethnicity were included to to denote inclusivity and understand the population of patients included. *ICD-10-PCS* defines major diagnostic or therapeutic operating room procedures.^17^ In-hospital mortality was defined by discharge disposition or an assigned *ICD-10-CM* code initiating transition to comfort care (Z51.5). Missingness of reported data were quantified (eTable 1 in Supplement 1).

The inpatient UPMC administrative and electronic health record (EHR) data were abstracted from the Clinical Data Warehouse including hospitalization demographics, with an uncapped number of potential *ICD-10-CM* codes.^18^ The inpatient data were supplemented with outpatient RAI-C scores computed at surgical clinics within 90 days of hospitalization.^10^

### Parameter Adaptation

The widely validated RAI is composed of 11 parameters and 2 statistical interactions.^12^ Age and sex were adapted to NIS variables. All pertinent *ICD-10-CM* codes were explored by 2 independent reviewers (A.D and Y.K.) for unintentional weight loss, poor appetite, congestive heart failure, shortness of breath, kidney failure, cancer, functional status (ie, level of independency), cognitive decline, and institutional living status at hospital admission.^19,20^ Malignant neoplasm codes (C00-C96) for cancer parameter definition were reviewed with additional scrutiny and categorized into severe, moderate, or mild based upon 5-year survival rates^21,22,23,24^ and were exclusive to active cancer diagnoses (eMethods, eTable 2, and eTable 3 in Supplement 1). The institutional living status parameter was omitted due to the absence of an applicable NIS variable or suitable *ICD-10-CM* code. All initial reviewer discrepancies were discussed among 3 additional reviewers (D.H., K.R., and C.B.) yielding consensus. For each hospitalization, the presence of more than 1 of the *ICD-10-CM* codes within each RAI parameter was interpreted as present.

### Statistical Analysis

All analyses were completed using Stata statistical software version 17 (StataCorp) and Prism version 9 (GraphPad) with code available .^25^ Data analysis occurred from January to May 2023.

#### Derivation, Validation, and Stratification

The RAI-ICD was NIS derived (2019) and validated (2020) using discharge data among inpatient hospitalizations for adults (≥18 years) undergoing major diagnostic or therapeutic operating room procedures. All NIS data analyses were survey weighted. Demographics, RAI-ICD parameters, and outcomes were presented as mean with standard error (SE) or proportion with 95% CI. Logistic regression using a robust sandwich estimator generated β coefficients among hospitalization data without missingness. Model selection among cancer categories was informed by discrimination and calibration estimated with C statistics and additional testing (eMethods in Supplement 1).

Logistic regression derived the RAI-ICD including 10 RAI parameters with 2 statistical interactions (age × cancer and functional status × cognitive decline) projecting in-hospital mortality. Mirroring the initial RAI-A derivation, the effect sizes (β coefficients) weighted each RAI parameter, generating an integerized RAI-ICD scoring system (range, 0-81 with a higher score indicating increased frailty).^9,12^

The integerized RAI-ICD scoring system was then validated (NIS 2020) among hospitalizations with major diagnostic or therapeutic operating room procedures. For each integer value of RAI-ICD, we calculated the observed and projected mortality, cumulative proportion of frailty, sensitivity, specificity, positive predictive value, negative predictive value, F1 score,^26^ and Matthews correlation coefficient (MCC).^27^ Projected mortality was computed with postprojection margins adjusting for the integerized RAI-ICD. The single best performing cancer categorization model was selected using model performance and testing parameters in combination with decision curve analysis. The RAI-ICD was then stratified into 4 categories of increasing mortality risk: robust, normal, frail, and very frail in accordance with methods previously established for RAI calibration (eTable 4 in Supplement 1).^12,13^ Secondary outcomes were compared across frailty categories and included elective admission, hospital length of stay, and hospital charges.

#### Sensitivity and Convergent Validity Analysis

Sensitivity analyses evaluating the external validity and result robustness included replicating the analysis in 2 alternative cohorts and testing an alternative mortality definition. The alternative cohorts included hospitalizations both with and without major diagnostic or therapeutic operating room procedures in the NIS (2020) and UPMC (2021-2022). The alterative in-hospital mortality definition included only the discharge disposition. Finally, we assessed convergent validity by Spearman rank correlation coefficients comparing the RAI-ICD with the RAI-C in the UPMC data as well as 2 alternative frailty indices in the NIS 2020: Hospital Frailty Risk Score (HFRS)^28^ and US Department of Veterans Affairs Frailty Index (VA-FI-10)^29^ modified to exclude *Current Procedural Terminology* (*CPT*) codes unavailable in the NIS.^30^

## Results

The estimated derivation population comprised 9 548 206 survey-weighted hospitalizations of patients (mean [SE] age, 55.4 [0.1] years; 3 742 330 males [weighted percentage, 39.8%; 95% CI, 38.8%-39.6%] and 5 804 431 females [weighted percentage, 60.8%; 95% CI, 60.4%-61.2%]; 1 136 237 Black individuals [weighted percentage, 11.9%; 95% CI, 11.3%-12.5%]; 6 445 039 White individuals [weighted percentage, 67.5%; 95% CI, 66.4-68.5]; and 1 413 134 individuals with another race or ethnicity [weighted percentage, 14.8%; 95% CI, 14.0%-15.7%]) (Table 1 and eFigure 1 in Supplement 1). It mirrored the primary survey-weighted validation population of 8 113 950 patients (mean [SE] age, 54.4 [0.1] years; 3 148 213 males [weighted percentage, 38.8%; 95% CI, 38.3%-39.3%] and 4 965 737 females [weighted percentage, 61.2%; 95% CI, 60.7%-61.7%]; 989 902 Black individuals [weighted percentage, 12.2%; 95% CI, 11.6%-12.8%]; 5 363 321 White individuals [weighted percentage, 66.1%; 95% CI, 65.1%-67.2%]; 1 265 776 individuals with another race or ethnicity [weighted percentage, 15.6%; 95% CI, 14.7%-16.4%]). Overall observed in-hospital mortality was 2.1% (95% CI, 2.1%-2.2%) for the derivation population and 2.5% (95% CI, 2.4%-2.5%) for the for the validation populations. Data missingness was less than 5% (eTable 5 in Supplement 1).

**Table 1.** Hospitalization Characteristics of Derivation and Validation Populations

Variable	Participants, No. (weighted %) [95% CI]	
	Derivation population: 2019 national inpatient sample (estimated N = 9 548 206)	Validation population: 2020 national inpatient sample (estimated N = 8 113 950)
Demographics		
Age, mean (SE) y	55.4 (0.1)	54.4 (0.1)
Sex		
Male	3 148 213 (39.2) [38.8-39.6]	3 742 330 (38.8) [38.3-39.3]
Female	4 965 737 (60.8) [60.4-61.2]	5 804 431 (61.2) [60.7-61.7]
Race and ethnicity		
Asian	315 091 (3.3) [3.0-3.6]	275 874 (3.4) [3.1-3.7]
Black	1 136 237 (11.9) [11.3-12.5]	989 902 (12.2) (11.6-12.8)
White	6 445 039 (67.5) (66.4-68.5]	5 363 321 (66.1) [65.1-67.2]
Other^a^	1 413 134 (14.8) [14.0-15.7)	1 265 776 (15.6) [14.7-16.4]
Missing^b^	238 705 (2.5) [2.1-3.0]	219 077 (2.7) [2.3-3.2]
Risk analysis index parameters		
Cancer^c^	410 572 (4.3) [4.0-4.5]	373 242 (4.6) [4.3-4.8]
Weight loss	105 030 (1.1) [1.1-1.2]	105 481 (1.3) [1.2-1.3]
Poor appetite	19 096 (0.2) [0.1-0.2]	81 140 (0.2) [0.2-0.2]
Kidney failure	372 380 (3.9) [3.8-4.0]	340 786 (4.2) [4.1-4.3]
Congestive heart failure	1 021 658 (10.7) [10.5-10.9]	933 104 (11.5) [11.2-11.7]
Shortness of breath	190 964 (2.0) [1.9-2.0]	154 165 (1.9) (1.8-1.9)
Cognitive decline	267 349 (2.8) [2.8-2.9]	243 419 (3.0) [3.0-3.0]
Functional status		
Independent	8 956 217 (93.8) [93.7-94.0]	7 602 771 (93.7) [93.6-93.9]
Partially dependent	467 862 (4.9) [4.9-5.0]	405 698 (5.0) [4.8-5.1]
Totally dependent	124 127 (1.3) [1.3-1.4]	105 481 (1.3) [1.3-1.4]
RAI score		
RAI-ICD integer, mean (SE)	20.7 (0.1)	20.3 (0.1)
RAI-ICD category		
Robust	6 559 618 (68.7) [68.3-69.1]	5 598 626 (69.0) [68.6-69.5]
Normal	1 966 930 (20.6) [20.2-20.8]	1 598 448 (19.7) [19.4-20.0]
Frail	630 182 (6.6) [6.5-6.8]	567 977 (7.0) [6.9-7.1]
Very frail	391 476 (4.1) [4.0-4.3]	348 899 (4.3) [4.1-4.4]

Abbreviation: RAI-ICD, Risk Analysis *Index–International Statistical Classification of Diseases*.

^a^ Race and ethnicity are categorized within National Inpatient Sample in 1 single data element. Other race and ethnicity includes Hispanic, Native American, and any other race or ethnicity not otherwise specified.

^b^ Reported if more than 0.1% of variables are missing.

^c^ Cancer variable is defined based on *International Classification of Diseases, Tenth Revision*, clinical codes for severe cancer categorization (eTable 3 in Supplement 1).

Of the approximately 68 000 available *ICD-10-CM* codes, 323 were selected for the final RAI-ICD (eTable 3 in Supplement 1). Together with age and sex, each adapted *ICD-10-CM* parameter definition and 2 interaction terms were weighted (eTable 6 in Supplement 1). The severe cancer categorization performed optimally (eResults, eFigure 2, and eTable 7 in Supplement 1) and constituted the final RAI-ICD cancer parameter definition, generating excellent derivation discrimination (C statistic, 0.810; 95% CI, 0.808-0.813). After weighting and integerizing the RAI-ICD (Table 2), the mean (SE) integerized RAI-ICD was 20.7 (0.1) for the derivation population and 20.3 (0.1) for the validation population. Observed mortality mirrored projected mortality, rising with increasing RAI-ICD score (Figure). The integerized RAI-ICD achieved good discrimination for in-hospital mortality (Table 3).

**Table 2.** RAI-ICD Parameter Weighting, Ranging From 0 to 81

Risk Analysis Index parameter	RAI-ICD weighting	
	Without cancer	With cancer
Age, y × cancer (with or without)^a^		
≤19	0	43
20-24	2	43
25-29	4	43
30-34	6	44
35-39	9	44
40-44	11	44
45-49	13	44
50-54	15	44
55-59	18	44
60-64	20	45
65-69	22	45
70-74	25	45
75-79	27	45
80-84	29	45
85-89	31	46
90-94	34	46
95-99	36	46
≥100	38	46
Male sex	3	3
Weight loss	2	2
Poor appetite	1	1
Kidney failure	3	3
Congestive heart failure	3	3
Shortness of breath	2	2
Functional status × cognitive decline (with or without)^a^		
Independent	Without cognitive decline, 0	With cognitive decline, 9
Partially dependent	Without cognitive decline, 10	With cognitive decline, 15
Totally dependent	Without cognitive decline, 20	With cognitive decline, 21

Abbreviation: RAI-ICD, Risk Analysis Index–*International Statistical Classification of Diseases, Tenth Revision, Clinical Modification*.

^a^ Statistical interaction term.

**Figure.**
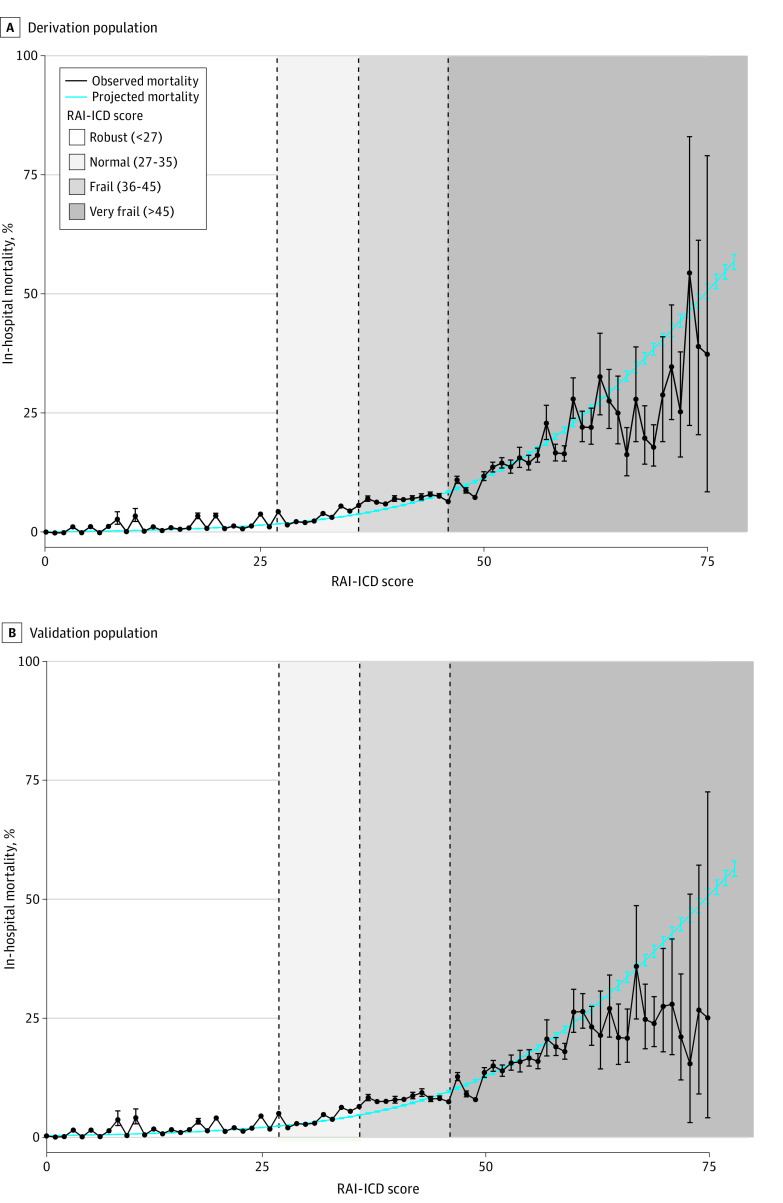
Observed and Projected Mortality by the Integerized Risk Analysis Index–*International Statistical Classification of Diseases, Tenth Revision, Clinical Modification* (RAI-ICD) Score Data are demonstrated among the derivation (National Inpatient Sample, 2019; Panel A) and validation (National Inpatient Sample, 2020; Panel B) populations including hospitalizations for major diagnostic or therapeutic operating room procedures. Line graphs with observed (black) and projected (blue) mortality connecting survey-weighted, population-based point estimates (dots) and associated 95% CIs (error bars) across the integerized RAI-ICD.

**Table 3.** RAI-ICD Performance

Analysis	C statistic (95% CI)
Primary: major diagnostic or therapeutic operating room procedures	
National inpatient sample, 2019 (derivation)	0.789 (0.787-0.791)
National inpatient sample, 2020 (validation)	0.784 (0.782-0.786)
Sensitivity: alternative definition of in-hospital mortality (national inpatient sample, 2020^a^	0.741 (0.738-0.743)
With and without major diagnostic or therapeutic operating room procedures	
National inpatient sample, 2020	0.778 (0.777-0.779)
UPMC, 2021-2022	0.860 (0.857-0.862)

Abbreviation: RAI-ICD, Risk Analysis Index–*International Statistical Classification of Diseases*.

^a^ In-hospital mortality exclusively defined by discharge disposition variable.

Observed and projected mortality, the cumulative proportion of the population, and performance statistics at each RAI-ICD integer are reported in eTable 4 in Supplement 1. Following methods established previously for the RAI-A and RAI-C,^13^ the RAI-ICD integer of 36 defined the threshold for frailty because projected mortality at this level (4.98%; 95% CI, 4.86%-5.09%) was approximately double that of the mortality observed within the validation population (2.48%; 95% CI, 2.42%-2.55%). This threshold also correlates with maximal F1 score (0.33) and MCC (0.26), representing an optimal balance between sensitivity and specificity, accuracy, and precision. Very frail scores (RAI-ICD >45) corresponded to mortality at least 4 times the overall observed mortality, whereas robust scores (RAI-ICD <27) reflected mortality below the overall observed mortality. Normal scores (RAI-ICD, 27-35) corresponded to observed mortalities between robust and frail (RAI-ICD, 36-45) (eTable 4 in Supplement 1). In-hospital mortality, emergent admissions, hospital length of stay, and total charges increased with increasing RAI-ICD frailty categorization (Table 4).

**Table 4.** In-Hospital Outcomes, Stratified by RAI-ICD Frailty Categorization in the National Inpatient Sample Validation Population

Analysis	RAI-ICD frailty categorization			
	Robust (<27)	Normal (27-35)	Frail (36-45)	Very frail (>45)
Primary analysis^a^				
Participants, estimated No. (weighted %)	5 592 500 (69.0)	1 598 695 (19.7)	565 760 (7.0)	345 225 (4.3)
Elective admission, % (95% CI)	49.78 (48.87-50.68)	43.72 (43.02-44.43)	30.31 (29.29-31.36)	34.78 (33.16-36.43)
In-hospital mortality, % (95% CI)	1.03 (0.99-1.08)	3.62 (3.52-3.71)	7.88 (7.66-8.10)	11.95 (11.55-12.36)
Length of hospital, mean (SE) d	4.40 (0.04)	6.28 (0.05)	8.21 (0.06)	8.80 (0.07)
Hospital charges, mean (SE), $	89 420.43 (1582.10)	138 684.90 (2238.85)	146 336.20 (2489.01)	151 211.10 (3182.51)
Sensitivity analysis^b^				
Participants, estimated No. (weighted %)	16 444 147 (59.5)	6 164 640 (22.3)	3 159 295 (11.4)	1 900 584 (6.8)
Elective admission, % (95% CI)	24.95 (24.35-25.56)	15.94 (15.57-16.33)	11.25 (10.87-11.65)	12.16 (11.60-12.74)
In-hospital mortality, % (95% CI)	1.99 (1.94-2.04)	6.87 (6.76-6.98)	12.86 (12.67-13.05)	21.25 (20.89-21.60)
Length of hospital stay, mean (SE) d	4.42 (0.03)	5.62 (0.03)	6.48 (0.03)	6.76 (0.04)
Hospital charges, mean (SE) $	59 582.34 (896.81)	77 989.05 (1106.00)	73 532.05 (1087.15)	79 217.37 (1458.88)

Abbreviation: RAI-ICD, Risk Analysis Index–*International Statistical Classification of Diseases*.

^a^ Hospitalizations with major diagnostic or therapeutic operating room procedures (estimated N = 8 113 950).

^b^ Hospitalizations with and without major diagnostic or therapeutic operating room procedures (estimated N = 2 766 866).

In sensitivity analysis, the alterative definition of mortality occurred among 126 724 NIS hospitalizations (1.6%), yielding good discrimination (C statistic, 0.741; 95% CI, 0.738-0.743) (Table 3). Hospitalizations with and without major diagnostic or therapeutic operating room procedures resulted in good discrimination for the 27 668 666 individuals in the NIS operative and nonoperative sample (C statistic, 0.778; 95% CI, 0.777-0.779) and the NIS operative sample (C statistic, 0.784; 95% CI, 0.782-0.786) and excellent discrimination for the 1 316 544 individuals in the UPMC sample (C statistic, 0.860; 95% CI, 0.857-0.862) (Table 3 and eTable 8 in Supplement 1). Outcome differentiation across the RAI-ICD integers and frailty categories mirrored that of the primary analysis (eFigure 3 in Supplement 1).

Exploration of convergent validity between the RAI-ICD, RAI-C, VA-FI-10, and HFRS demonstrated similar discrimination (C statistic for RAI-C, 0.814 [95% CI, 0.809-0.819]; C statistic for HFRS, 0.868 [95% CI, 0.867-0.870]; and C statistic for VA-FI-10, 0.758 [95% CI, 0.756-0.760]). The RAI-ICD and the VA-FI-10 were strongly correlated (ρ, 0.701; 95% CI, 0.700-0.702), but the HFRS and RAI-ICD (ρ, 0.560; 95%CI, 0.559-0.561) and HFRS and VA-FI-10 (ρ, 0.669; 95% CI, 0.669-0.670) were moderately correlated (eTable 9 and eFigure 4 in Supplement 1).

## Discussion

In this cohort study, we successfully adapted, derived, stratified, and validated the RAI frailty score using *ICD-10-CM* codes in a population estimate including millions of contemporary US hospitalizations. The RAI-ICD was systematically developed using a broad range of *ICD-10-CM* codes that were carefully adapted to previously validated parameters defining frailty.^12^ In both the NIS population and a large multihospital system cohort, the RAI-ICD achieved excellent discrimination for in-hospital mortality with increasing frailty and was associated with increasing hospital resource utilization. The performance of the RAI-ICD was comparable with previously validated RAI iterations (C statistic range, 0.77-0.86).^9,12,13^ These data support and further strengthen the RAI as a robust and versatile frailty assessment tool among operative and nonoperative patient populations that may now be applied to any dataset including *ICD-10* codes.

Frailty affects millions of older adults worldwide, generating not only an increased risk of adverse patient outcomes but also contributing to ever-growing health care expenditures. For example, while representing nearly 4% of the US Medicare population, frail individuals were responsible for nearly one-half of the overall potentially preventable high cost spendings.^5^ In the NIS, which includes all payers, the RAI-ICD again demonstrates frailty was associated with both increasing in-hospital mortality and resource utilization.

Prospectively applied, validated, and survey-based assessment tools identifying frail patients have demonstrated that targeted interventions (eg, nutritional, social, and physical support) may improve outcomes.^8^ However, consensus on a precise definition of frailty remains elusive,^31^ and apart from the Comprehensive Geriatric Assessment requiring 60 to 90 minutes of face-to-face time with a geriatrician specialist, no single benchmark frailty assessment has emerged. The resulting proliferation of frailty tools and ongoing disagreement about the underlying conceptual models led a recent National Institute of Health consensus panel^32^ to recommend using existing measures whenever possible, moving beyond conceptual disagreements, and focusing on clinical practice. We agree and offer the RAI-ICD as an extension of the existing RAI-A and RAI-C, applying a uniform conceptual model to a wide variety of clinical contexts. The RAI-ICD is applicable to many publicly available and EHR datasets for cross-disciplinary, retrospective investigation. Furthermore, the findings from such investigations can be immediately implemented at the bedside using the prospective, widely validated RAI-C survey with proven feasibility in as little as 30 seconds.^11^

The RAI-ICD is not the only claims-based frailty tool utilizing *ICD-10-CM* codes, and should be considered in context with alternatives such as the HFRS^28^ and the claims-based frailty indices (CFI) by Kim et al (CFI-Kim)^33^ and Segal et al (CFI-Segal),^34^ along with the Orkaby et al^19^ CFI for use among US military veterans (VA-FI-10). Each of these frailty indices demonstrates similar model discrimination even as they tend to identify frailty among differing sets of patients with only modest intersection.^12,35^ As such, the choice of any given tool depends less on model performance and more on the tool’s conceptual validity, interpretability, and feasibility.

Each of the aforementioned *ICD-10-CM*–based frailty assessments were informed by existing conceptual models of frailty, meeting a baseline threshold of face validity. Frailty-related ranges of administrative diagnostic and procedural codes were selected based on expert opinion alone (HFRS) or mapping to either the Rockwood cumulative deficit (RAI-ICD, CFI-Kim, and VA-FI-10) or frailty phenotype (CFI-Segal) conceptual models.^28,36,37,38^ However, the initial, broad, clinician-guided selection of codes for the HFRS, CFI-Kim, and CFI-Segal tools were then tapered down and finalized using black-box, machine-learning techniques. Because the Kim, Segal and Orkaby CFIs include approximately 27 000, 2600, and 7000 *ICD-10* codes, respectively, a data-driven approach is necessary. On close inspection, some included codes are unrelated to frailty and thus conceptually inappropriate. For example, the HRFS relies heavily on acute diagnoses (eg, acute kidney failure or hypotension); the CFI-Kim includes infectious diseases (eg, pneumonia and influenza), lacerations, and paternity tests^33^; and both the CFI-Segal and VA-FI include comorbidities (eg, hypertension and hyperlipidemia) highly prevalent in robust populations and not particular to frailty. As such, the face validity of these tools can be challenged, and these inconsistencies may explain their poor performance in specific disease states and among those that are critically ill.^39,40^ Furthermore, the excellent discrimination of the HRFS for in-hospital mortality in the NIS may, in fact, highlight the tool’s selection of highly morbid, acute conditions without necessarily demonstrating specificity for frailty. For example, a 25-year-old professional athlete who slips on their stairs at home and falls again upon getting up with a resulting concussion and small traumatic intracranial hemorrhage would obtain an HFRS score consistent with frailty despite being an elite athlete (eMethods in Supplement 1). Finally, although the originating authors of the HFRS caution it is validated only for patients older than 75 years and is not intended for individualized clinical patient decision-making,^41,42^ growing literature applies it for precisely these purposes.^43,44,45,46^

Across all *ICD-10-CM*–based frailty tools, one potential advantage is the capacity to automate and embed frailty calculations within an EHR. Operationalizing is not trivial^47,48,49^; however, automated frailty assessment could facilitate efficient, prospective, population-level assessments,^28^ including EHR-embedded pragmatic trials promising for both containment of ever-rising trial costs^50,51,52,53,54^ and improving outcomes in efficient, self-learning health care systems.^55,56^ Notably, such automaticity is possible only for patients with previously documented diagnoses. Therefore, an appropriate frailty designation will both change fluidly with care and may misclassify a robust status among the most isolated and, therefore, vulnerable patients without prior exposure to the health care system for diagnoses. Therefore, automated code-based assessments must be supplemented by alterative, rapid, and feasible bedside assessment. The prospectively generated RAI-C is one such assessment,^11^ successfully embedded in Epic,^57^ Cerner,^58^ and the VA Computerized Patient Record System^59^ EHRs.

The applicability of each *ICD-10-CM*–based tool has limits. First, the HFRS (Hospital Episode Statistics database, >75 years), ICF-Kim (Cardiovascular Health Study, >65 years),^33^ ICF-Segal (Medicare beneficiaries),^34^ and VA-FI-10 (Veterans Health Association, >75 years)^19^ were all derived and validated in datasets including exclusively older cohorts, limiting their application. While age and frailty often correlate, they are independent. Elderly patients can be robust, and young patients can be frail. Second, the data elements required to compute the index delineate its applicability. CFI-Kim (*CPT* and Healthcare Common Procedure Coding System), CFI-Segal (history of prior admission), VA-FI-10 (*CPT*) all include data elements beyond demographic data and *ICD-10* codes. Simultaneously leveraging multiple code catalogs (ie, *ICD-10* or *CPT*) may theoretically increase model performance,^60,61^ but at a cost; not all datasets contain the code catalogs required to compute these indices, again limiting their application. Furthermore, the theoretical performance improvement of including codes across catalogs has not yet demonstrated clear benefit. For example, the NIS does not contain *CPT* codes, forcing their omission from VA-FI-10 scoring here. And yet, the VA-FI-10 generated good discrimination and convergent validity with the RAI-ICD, suggesting the increased complexity of numerous coding catalogs may not be required. Finally, CFI-Segal has not yet been translated from *International Classification of Diseases, Ninth Revision, Clinical Modification (ICD-9-CM) *to *ICD-10-CM*, highlighting the need to continuously adapt and recalibrate tools.

Common to all *ICD-9-CM* and *ICD-10-CM* based frailty assessments are 2 additional limitations. First, the number of data elements available within datasets varies. For example, the maximal number of *ICD-9-CM* and *ICD-10-CM* codes recorded for each hospitalization is 20 for the Hospital Episode Statistics database, 40 for the NIS, and unlimited for the UPMC EHR. This heterogeneity may alter model performance across datasets, introducing bias. Second, *ICD-9-CM* and *ICD-10-CM* coding assignments are notorious sources of inaccuracy with omission of granular details and unwarranted variability across clinicians, coders, hospital-systems, regions, and time. As a result, highly utilized, accurate codes in one setting or era may be omitted or misused elsewhere. Although these problems reduced over time,^62,63,64^ they are especially prevalent for *ICD-10-CM* Z-codes. They allow for inclusion of previously omitted frailty concepts including functional capacity. Specifically, they represent factors associated with health status that have contact with and dependence on the health care system, which conceptually match to frailty such as problems related to life-management difficulty.^28,65^ Although payers and clinicians alike promoted Z-code use, they remain both vastly underutilized and their application varies by patient factors (eg, gender and race), hospitals (eg, location and teaching status), and geography.^65^

### Limitations

This study has limitations specific to the RAI-ICD. First, the RAI-ICD was optimized and validated exclusively from retrospectively obtained, in-hospital data in which the characteristics of the validation cohort mirrored the derivation cohort, potentially limiting generalizability. Second, although we tested the RAI-ICD in multiple datasets, further evaluation of generalizability is required. Third, the complete *ICD-10-CM* code review was completed by 2 authors (A.D. and Y.K.) with discrepancies discussed among three additional authors (D.H., K.R., and C.B.) with an iterative focus on maximizing specificity; however, interrater reliability was not recorded. Fourth, in optimizing test statistics, the RAI-ICD was highly specific but less sensitive, minimizing the misclassification of nonfrail patients as frail; however, the RAI-ICD may warrant further context specific calibration (eg, screening) if greater sensitivity is required.

## Conclusion

In this cohort study, when the RAI parameters were adapted to the *ICD-10-CM*, increasing RAI-ICD scores were associated with an increase in hospital length of stay, hospital charges, and in-hospital mortality. With over 60 frailty indices available, each conceptually unique and applicable to disparate datasets, the main benefit of the RAI-ICD is that it extends the quantification of frailty to datasets with access to administrative data including *ICD-10-CM* codes using a unified conceptual framework validated in both prospective and retrospective applications.

## Notes

Supplement 1. **eTable 1.** Data Elements and Specific Variables from National Inpatient Sample
eMethods.
**eTable 2.** Adaptation of Cancer Categorizations Into Clinically Comparable *ICD-10-CM* Codes and Corresponding Remission Codes **eTable 3.** Finalized Adaptation of the Risk Analysis Index Parameters Into Clinically Comparable *ICD-10* Codes **eTable 4.** Final RAI-ICD Thresholds and Measures of Diagnostic Accuracy for Inpatient Mortality as Calibrated in the Validation National Inpatient Sample Population (2020) **eFigure 1.** Survey-Weighted and Estimated Populations in National Inpatient Sample (NIS) **eTable 5.** Data Missingness in Operative National Impatient Populations Among Adult Hospitalizations **eTable 6.** Logistic Regression for In-Hospital Mortality Weighting Risk Analysis Index Parameters in the National Inpatient Sample Derivation Population (2019)
eResults.
**eFigure 2.** Decision Curve Analysis for RAI-ICD for Alterative Cancer Parameter Definitions **eTable 7.** Model Performance Based on *ICD-10-CM* Codes for Each Cancer Categorization in National Inpatient Sample Derivation Population (2019) **eTable 8.** Baseline Characteristics of Sensitivity Cohorts Including Operative and Non-Operative Hospitalizations **eFigure 3.** Observed and Predicted Mortality by the Integerized RAI-ICD Score for Sensitivity Analysis **eTable 9.** Spearman Correlation Between Various Frailty Tools **eFigure 4.** Distribution of Frailty Categorization Among UPMC Hospitalizations with an Available Outpatient Risk Analysis Index – Clinical (RAI-C) Score
eReferences.

Supplement 2.
Data Sharing Statement
