# Supplementary material for: Adaptation of the Risk Analysis Index for Frailty Assessment Using Diagnostic Codes
Source: JAMA Netw Open. 2024 May 24;7(5):e2413166. doi: 10.1001/jamanetworkopen.2024.13166 (PMC11127118; doi:10.1001/jamanetworkopen.2024.13166)
Supplement: Supplement 1. — eTable 1. Data Elements and Specific Variables from National Inpatient Sample eMethods. eTable 2. Adaptation of Cancer Categorizations Into Clinically Comparable ICD-10-CM Codes and Corresponding Remission Codes eTable 3. Finalized Adaptation of the Risk Analysis Index Parameters Into Clinically Comparable ICD-10 Codes eTable 4. Final RAI-ICD Thresholds and Measures of Diagnostic Accuracy for Inpatient Mortality as Calibrated in the Validation National Inpatient Sample Population (2020) eFigure 1. Survey-Weighted and Estimated Populations in National Inpatient Sample (NIS) eTable 5. Data Missingness in Operative National Impatient Populations Among Adult Hospitalizations eTable 6. Logistic Regression for In-Hospital Mortality Weighting Risk Analysis Index Parameters in the National Inpatient Sample Derivation Population (2019) eResults. eFigure 2. Decision Curve Analysis for RAI-ICD for Alterative Cancer Parameter Definitions eTable 7. Model Performance Based on ICD-10-CM Codes for Each Cancer Categorization in National Inpatient Sample Derivation Population (2019) eTable 8. Baseline Characteristics of Sensitivity Cohorts Including Operative and Non-Operative Hospitalizations eFigure 3. Observed and Predicted Mortality by the Integerized RAI-ICD Score for Sensitivity Analysis eTable 9. Spearman Correlation Between Various Frailty Tools eFigure 4. Distribution of Frailty Categorization Among UPMC Hospitalizations with an Available Outpatient Risk Analysis Index – Clinical (RAI-C) Score eReferences. [file jamanetwopen-e2413166-s001.pdf]

Supplemental Online Content

Dicpinigaitis AJ, Khamzina Y, Hall DE, et al. Adaptation of the risk analysis index for frailty assessment using diagnostic codes. *JAMA Netw Open*. 2024;7(5):e2413166. doi:10.1001/jamanetworkopen.2024.13166

eTable 1. Data Elements and Specific Variables from National Inpatient Sample
eMethods.
eTable 2. Adaptation of Cancer Categorizations Into Clinically Comparable *ICD-10-CM* Codes and Corresponding Remission Codes
eTable 3. Finalized Adaptation of the Risk Analysis Index Parameters Into Clinically Comparable *ICD-10* Codes
eTable 4. Final RAI-ICD Thresholds and Measures of Diagnostic Accuracy for Inpatient Mortality as Calibrated in the Validation National Inpatient Sample Population (2020)
eFigure 1. Survey-Weighted and Estimated Populations in National Inpatient Sample (NIS)
eTable 5. Data Missingness in Operative National Impatient Populations Among Adult Hospitalizations
eTable 6. Logistic Regression for In-Hospital Mortality Weighting Risk Analysis Index Parameters in the National Inpatient Sample Derivation Population (2019)
eResults.
eFigure 2. Decision Curve Analysis for RAI-ICD for Alterative Cancer Parameter Definitions
eTable 7. Model Performance Based on *ICD-10-CM* Codes for Each Cancer Categorization in National Inpatient Sample Derivation Population (2019)
eTable 8. Baseline Characteristics of Sensitivity Cohorts Including Operative and Non-Operative Hospitalizations
eFigure 3. Observed and Predicted Mortality by the Integerized RAI-ICD Score for Sensitivity Analysis
eTable 9. Spearman Correlation Between Various Frailty Tools
eFigure 4. Distribution of Frailty Categorization Among UPMC Hospitalizations with an Available Outpatient Risk Analysis Index – Clinical (RAI-C) Score

This supplemental material has been provided by the authors to give readers additional information about their work.

© 2024 Dicpinigaitis AJ et al. *JAMA Network Open.*

eTable 1. Data Elements and Specific Variables from National Inpatient Sample

National Inpatient Sample variable	National Inpatient Sample descriptor
AGE	Age in years at admission
FEMALE	Indicator of sex
RACE	Race and ethnicity of patient[†]
DIED	Died during hospitalization
I10_DXn	ICD-10-CM Diagnosis
PCLASS_ORPROC	Major operating room ICD-10-PCS procedure indicator
DISCWT[‡]	Weight to discharges in the universe
HOSP_NIS	NIS hospital number
ELECTIVE	Elective versus non-elective admission
LOS	Length of stay, cleaned
TOTCHG	Total charges, cleaned
YEAR	Calendar year

International Classification of Diseases, Tenth Revision, Clinical Modification (ICD-10-CM)
ICD-10 Procedural Coding System (ICD-10-PCS)
National Inpatient Sample (NIS)
[†] Race and ethnicity within National Inpatient Sample are categorized in one single data element
[‡] is the discharge-level weight on the Healthcare Cost and Utilization Project (HCUP) nationwide databases, it is used to produce national estimates
NIS descriptors are presented as their appear on HCUP website, additional information regarding data elements and variables can be found on https://hcup-us.ahrq.gov/db/nation/nis/nisdde.jsp

© 2024 Dicpinigaitis AJ et al. *JAMA Network Open.*

eMethods.

Codes selected for the Risk Analysis Index (RAI)

All *International Classification of Diseases, Tenth Revision, Clinical Modification* (ICD-10-CM) codes adapted to RAI parameters were meticulously selected by clinician authors using online ICD-10-CM tools and supported by previously published literature. [1-3]

Special attention was paid to the definition of the cancer parameter. All available ICD-10-CM codes for categorized as neoplasm (C00-D99) were screened for potential inclusion by authors. [4] Only malignant neoplasms (C00-C96) were selected for further parameter definition. ‘Other’ and ‘unspecified’ malignancies, Kaposi’s sarcoma, non-melanoma skin cancers, and malignant neoplasms of placenta were excluded from further analysis. For the remaining neoplasm ICD-10-CM codes, the corresponding five-years survival was determined from publicly available data (https://seer.cancer.gov/statfacts/). [5, 6] These codes were divided into three groups: severe cancer with an estimate of ≤ 33% five-year survival rate, moderate cancer with a 34-67% five-year survival rate, and mild cancer with a ≥67% five-year survival rate.

We then used Z-codes indicating cancer in remission (Z85 ICD-10-CM codes). Hospitalizations with coexisting Z and C codes for the same pathology were defined as having a remission and were recategorized as not having active cancer in the final parameter definition. For example, a hospitalization with a C-code indicating esophageal cancer (e.g., C153) and a concurrent Z-code indicating esophageal cancer remission (e.g., Z8501) was not defined as having active cancer and therefore the coding pair did not yield identification of the cancer RAI-ICD parameter. In cases of leukemias, in addition to concurrent Z-codes, we included only the C-codes that specified ‘not having achieved remission’ or ‘in relapse’ to ensure the codes indicated an active cancer not in remission.

The optimally behaving cancer definition (i.e., mild, moderate, or severe) was selected based upon optimal C-statistics and Decision Curve Analysis (DCA). Notably, commonly applied Stata 17.0 macros for model testing are not functionally applicable to the sample weighting required for accurate use of the National Inpatient Sample (NIS). Therefore, model selection was supported further by Aikake Information Criteria (AIC), [7] Bayesian Information Criteria (BIC) [8] in the unweighted sample and “overlap” defined as the proportion of cases where the 95% confidence interval (CI) or the observed mortality included the predicted mortality. [9] To calculate the C-statistic on the weighted NIS population, phat was predicted for the weighted model and subsequently the Roger Newson’s programs ‘somersd’ (available at SSC) computed the weighted models AUC based on predicted mortality of the population.

Sample code:

```
svy: logistic died rai_severe if age>=18 & pclass_orproc==1, or nolog  // survey weighted model
predict phat

somersd died phat [pweight=discwt] if age>=18 & pclass_orproc==1, tr(c)
matrix b = e(b)
local auc = b[1,1]

di   "Area under the Curve: " %6.5f `auc'
```

Summation of HRFS in discussion:
W19 “unspecified fall” (3.2 points), SOO “Superficial Injury of the Head” (3.2), R41 “Symptoms/Signs involving cognitive functions and awareness” (2.7), SO6 “intracranial injury” (2.4), & R29 “repeated falls” (3.6). The total score of 15.1 would cause the patient to be classified as “frail” despite being an elite professional athlete who is as robust as possible with the maximum possible physiologic reserve.

© 2024 Dicpinigaitis AJ et al. *JAMA Network Open.*

eTable 2. Adaptation of Cancer Categorizations Into Clinically Comparable *ICD-10-CM* Codes and Corresponding Remission Codes

Cancer categorization	Malignant Code Descriptor	Malignant neoplasm ICD-10-CM codes	Remission ICD-10-CM code(s)
Severe cancer (5-year all-cause mortality >67%)	Malignant neoplasm of esophagus	C153, C154, C155, C158, C159	Z8501
	Malignant neoplasm of liver and intrahepatic bile ducts	C220, C221, C222, C223, C224, C227, C228, C229	Z8505
	Malignant neoplasm of gallbladder	C23	
	Malignant neoplasm of other and unspecified parts of biliary tract	C240, C241, C248, C249	
	Malignant neoplasm of pancreas	C250, C251, C252, C253, C254, C257, C258, C259	Z8507
	Malignant neoplasm of trachea	C33	Z8512
	Malignant neoplasm of bronchus and lung	C3400, C3401, C3402, C3410, C3411, C3412, C342, C3430, C3431, C3432, C3480, C3481, C3482, C3490, C3491, C3492	Z85118
	Mesothelioma	C450, C451, C452, C457, 459	
	Malignant neoplasm of brain	C710, C711, C712, C713, C714, C715, C716, C717, C718, C719	Z85841
	Secondary and unspecified malignant neoplasm of lymph nodes	C770, C771, C772, C773, C774, C775, C778, C779	
	Secondary malignant neoplasm of respiratory and digestive organs	C7800, C7801, C7802, C781, C782, C7830, C7839, C784, C785, C786, C787, C7880, C7889	
	Secondary malignant neoplasm of other and unspecified sites	C7900, C7901, C7902, C7910, C7911, C7919, C792, C7931, C7932, C7940, C7949, C7951, C7952, C7960, C7961, C7962, C7963, C7970, C7971, C7972, C7981, C7982, C7989, C799	
	Disseminated malignant neoplasm, unspecified	C800	
	Secondary neuroendocrine tumors	C7B00, C7B01, C7B02, C7B03, C7B04, C7B09, C7B1, C7B8	
	Acute myeloblastic leukemia; Acute myeloid leukemia with 11q23-abnormality; Acute myeloid leukemia with multilineage dysplasia[†]	C9200, C9202, C9260, C9262, C92A0, C92A2	Z856
	Monocytic leukemia[†]	C9300, C9302, C9310, C9312, C9330, C9332, C93Z0, C93Z2, C9390, C9392	Z856

© 2024 Dicpinigaitis AJ et al. *JAMA Network Open.*

**Cancer categorization** ***Continued***	**Malignant Code Descriptor** ***Continued***	**Malignant neoplasm ICD-10-CM codes** ***Continued***	**Remission ICD-10-CM code(s)** ***Continued***
Moderate cancer (5-year all-cause mortality 34-67%)	Malignant neoplasm of gum	C030, C031, C039	Z85818
	Malignant neoplasm of floor of mouth	C040, C041, C048, C049	Z85818
	Malignant neoplasm of hard palate	C050	Z85818
	Malignant neoplasm of stomach	C160, C161, C162, C163, C164, C165, C166, C168, C169	Z85028
	Malignant neoplasm of colon	C180, C181, C182, C183, C184, C185, C186, C188, C189	Z85038
	Malignant neoplasm of rectosigmoid junction	C19	Z85048
	Malignant neoplasm of nasal cavity and middle ear	C300, C301	Z8522
	Malignant neoplasm of accessory sinuses	C310, C311, C312, C313, C318, C319	Z8522
	Malignant neoplasm of larynx	C320, C321, C322, C323, C328, C329	Z8521
	Malignant neoplasm of heart, mediastinum and pleura	C380, C381, C382, C383, C384, C388	
	Merkel cell carcinoma	C4A0, C4A10, C4A111, C4A112, C4A121, C4A122, C4A20, C4A21, C4A22, C4A30, C4A31, C4A39, C4A4, C4A51, C4A52, C4A59, C4A60, C4A61, C4A62, C4A70, C4A71, C4A72, C4A8, C4A9	Z85821
	Malignant neoplasm of peripheral nerves and autonomic nervous system	C470, C4710, C4711, C4712, C4720, C4721, C4722, C473, C474, C475', C476, C478, C479	Z85848
	Malignant neoplasm of other connective and soft tissue	C490, C4910, C4911, C4912, C4920, C4921, C4922, C493, C494, C495, C496, C498, C499, C49A0, C49A1, C49A2, C49A3, C49A4, C49A5, C49A9	Z85831
	Malignant neoplasm of vagina	C52	Z8544
	Malignant neoplasm of cervix uteri	C530, C531, C538, C539	Z8541
	Malignant neoplasm of ovary	C561, C562, C563, C569	Z8543
	Malignant neoplasm of penis	C600, C601, C602, C608, C609	
	Malignant neoplasm of renal pelvis	C651, C652, C659	Z8553
	Malignant neoplasm of meninges	C700, C701, C709	Z85848

© 2024 Dicpinigaitis AJ et al. *JAMA Network Open.*

	**Malignant Code Descriptor** ***Continued***	**Malignant neoplasm ICD-10-CM codes** ***Continued***	**Remission ICD-10-CM code(s)** ***Continued***
	Malignant neoplasm of spinal cord, cranial nerves and other parts of central nervous system	C720, C721, C7220, C7221, C7222, C7230, C7231, C7232, C7240, C7241, C7242, C7250, C7259, C729	Z85848
	Malignant neoplasm of adrenal gland	C7400, C7401, C7402, C7410, C7411, C7412, C7490, C7491, C7492	Z85858
	Malignant neuroendocrine tumors	C7A00, C7A010, C7A011, C7A012, C7A019, C7A020, C7A021, C7A022, C7A023, C7A024, C7A025, C7A026, C7A029, C7A090, C7A091, C7A092, C7A093, C7A094, C7A095, C7A096, C7A098, C7A1, C7A8	Z85020, Z85030, Z85040, Z85060, Z85110, Z85230, Z85520
	Mature T/NK-cell lymphomas	C8400, C8401, C8402, C8403, C8404,C8405, C8406, C8407, C8408, C8409,C8410, C8411, C8412, C8413, C8414,C8415, C8416, C8417, C8418, C8419,C8440, C8441, C8442, C8443, C8444,C8445, C8446, C8447, C8448, C8449,C8460, C8461, C8462, C8463, C8464,C8465, C8466, C8467, C8468, C8469,C8470, C8471, C8472, C8473, C8474,C8475, C8476, C8477, C8478, C8479,C847A,C84A0, C84A1, C84A2, C84A3, C84A4,C84A5, C84A6, C84A7, C84A8, C84A9,C84Z0, C84Z1, C84Z2, C84Z3, C84Z4,C84Z5, C84Z6, C84Z7, C84Z8, C84Z9,C8490, C8491, C8492, C8493, C8494,C8495, C8496, C8497, C8498, C8499	Z8572
	Multiple myeloma and malignant plasma cell neoplasms	C9000, C9002, C9010, C9012, C9020, C9022, C9030, C9032	Z8579
Mild cancer (5-year all-cause mortality ≤33%)	Malignant neoplasm of lip	C000, C001, C002, C003, C004, C005, C006, C008, C009	Z85818
	Malignant neoplasm of base of tongue	C01	Z85810
	Malignant neoplasm of other and unspecified parts of tongue	C020, C021, C022, C023, C024, C028, C029	Z85810
	Malignant neoplasm of soft palate	C051, C052, C058, C059	Z85818

© 2024 Dicpinigaitis AJ et al. *JAMA Network Open.*

	Malignant Code Descriptor *Continued*	Malignant neoplasm ICD-10-CM codes *Continued*	Remission ICD-10-CM code(s) *Continued*
	Malignant neoplasm of parotid gland	C07	Z85818
	Malignant neoplasm of other and unspecified major salivary glands	C080, C081, C089	Z85818
	Malignant neoplasm of tonsil	C090, C091, C098, C099	Z85818
	Malignant neoplasm of oropharynx	C100, C101, C102, C103, C104, C108, C109	Z85818
	Malignant neoplasm of nasopharynx	C110, C111, C112, C113, C118, C119	Z85818
	Malignant neoplasm of pyriform sinus	C12	Z85818
	Malignant neoplasm of hypopharynx	C130, C131, C132, C138, C139	Z85818
	Malignant neoplasm of other and ill-defined sites in the lip, oral cavity, and pharynx	C140, C142, C148	Z85818
	Malignant neoplasm of small intestine	C170, C171, C172, C173, C178, C179	Z85068
	Malignant neoplasm of rectum	C20	Z85048
	Malignant neoplasm of anus and anal canal	C210, C211, C212, C218	Z85048
	Malignant neoplasm of thymus	C37	Z85238
	Malignant neoplasm of bone and articular cartilage of limbs	C4000, C4001, C4002, C4010, C4011, C4012, C4020, C4021, C4022, C4030, C4031, C4032, C4080, C4081, C4082, C4090, C4091, C4092	Z85830
	Malignant neoplasm of bone and articular cartilage of other and unspecified sites	C410, C411, C412, C413, C414, C419	Z85830
	Malignant melanoma of skin	C430, C4310, C43111, C43112, C43121, C43122, C4320, C4321, C4322, C4330, C4331, C4339, C434, C4351, C4352, C4359, C4360, C4361, C4362, C4370, C4370, C4371, C4372, C438, C439	Z85820
	Malignant neoplasm of breast	C50011, C50012, C50019, C50021, C50022, C50029, C50111, C50112, C50119, C50121, C50122, C50129, C50221, C50222, C50229, C50311, C50312, C50319, C50321, C50322, C50329, C50411, C50412, C50419, C50421, C50422, C50429, C50511, C50512, C50519, C50521, C50522, C50529, C50611, C50612, C50619, C50621, C50622, C50629, C50811, C50812, C50819, C50821, C50822, C50829, C50911, C50912, C50919, C50921, C50922, C50929	Z853

© 2024 Dicpinigaitis AJ et al. *JAMA Network Open.*

	Malignant neoplasm ICD-10-CM codes *Continued*	Remission ICD-10-CM code(s) *Continued*	Malignant Code Descriptor *Continued*
	Malignant neoplasm of vulva	C510, C511, C512, C518, C519	Z8544
	Malignant neoplasm of corpus uteri	C540, C541, C542, C543, C548, C549	Z8542
	Malignant neoplasm of uterus, part unspecified	C55	Z8542
	Malignant neoplasm of prostate	C61	Z8546
	Malignant neoplasm of testis	C6200, C6201, C6202, C6210, C6211, C6212, C6290, C6291, C6292	Z8547
	Malignant neoplasm of kidney, except renal pelvis	C641, C642, C649	Z85528
	Malignant neoplasm of ureter	C661, C662, C669	Z8554
	Malignant neoplasm of bladder	C670, C671, C672, C673, C674, C675, C676, C677, C678, C679	Z8551
	Malignant neoplasm of eye and adnexa	C6900, C6901, C6902, C6910, C6911, C6912, C6920, C6921, C6922, C6930, C6931, C6932, C6940, C6941, C6942, C6950, C6951, C6952, C6960, C6961, C6962, C6980, C6981, C6982, C6990, C6991, C6992	Z85840
	Malignant neoplasm of thyroid gland	C73	Z85850
	Hodgkin lymphoma	C8100, C8101, C8102, C8103, C8104, C8105, C8106, C8107, C8108, C8109, C8110, C8111, C8112, C8113, C8114, C8115, C8116, C8117, C8118, C8119, C8120, C8121, C8122, C8123, C8124, C8125, C8126, C8127, C8128, C8129, C8130, C8131, C8132, C8133, C8134, C8135, C8136, C8137, C8138, C8139, C8140, C8141, C8142, C8143, C8144, C8145, C8146, C8147, C8148, C8149, C8170, C8171, C8172, C8173, C8174, C8175, C8176, C8177, C8178, C8179, C8190, C8191, C8192, C8193, C8194, C8195, C8196, C8197, C8198, C8199	Z8571

	**Malignant Code Descriptor** *Continuation*	**Malignant neoplasm ICD-10-CM codes** *Continuation*	**Remission ICD-10-CM code(s)** *Continuation*
	Follicular lymphoma	C8200, C8201, C8202, C8203, C8204, C8205, C8206, C8207, C8208, C8209, C8210, C8211, C8212, C8213, C8214, C8215, C8216, C8217, C8218, C8219, C8220, C8221, C8222, C8223, C8224, C8225, C8226, C8227, C8228, C8229, C8230, C8231, C8232, C8233, C8234, C8235, C8236, C8237, C8238, C8239, C8240, C8241, C8242, C8243, C8244, C8245, C8246, C8247, C8248, C8249, C8250, C8251, C8252, C8253, C8254, C8255, C8256, C8257, C8258, C8259, C8260, C8261, C8262, C8263, C8264, C8265, C8266, C8267, C8268, C8269, C8280, C8281, C8282, C8283, C8284, C8285, C8286, C8287, C8288, C8289, C8290, C8291, C8292, C8293, C8294, C8295, C8296, C8297, C8298, C8299	Z8572
	Non-follicular lymphoma	C8300, C8301, C8302, C8303, C8304, C8305, C8306, C8307, C8308, C8309, C8310, C8311, C8312, C8313, C8314, C8315, C8316, C8317, C8318, C8319, C8330, C8331, C8332, C8333, C8334, C8335, C8336, C8337, C8338, C8339, C8350, C8351, C8352, C8353, C8354, C8355, C8356, C8357, C8358, C8359, C8370, C8371, C8372, C8373, C8374, C8375, C8376, C8377, C8378, C8379, C8380, C8381, C8382, C8383, C8384, C8385, C8386, C8387, C8388, C8389, C8390, C8391, C8392, C8393, C8394, C8395, C8396, C8397, C8398, C8399	Z8572
	Other specified and unspecified types of non-Hodgkin lymphoma	C8510, C8511, C8512, C8513, C8514, C8515, C8516, C8517, C8518, C8519, C8520, C8521, C8522, C8523, C8524, C8525, C8526, C8527, C8528, C8529, C8350, C8351, C8352, C8353, C8354, C8355, C8356, C8357, C8358, C8359, C8580, C8581, C8582, C8583, C8584, C8585, C8586, C8587, C8588, C8589, C8590, C8591, C8592, C8593, C8594, C8595, C8596, C8597, C8598, C8599	Z8572
	Lymphoid leukemia†	C9100, C9102, C9110, C9112, C9130, C9132, C9140, C9142, C9150, C9152, C9160, C9162, C91A0, C91A2, C91Z0, C91Z2, C9190, C9192	Z856

© 2024 Dicpinigaitis AJ et al. *JAMA Network Open.*

	**Malignant Code Descriptor** *Continuation*	**Malignant neoplasm ICD-10-CM codes** *Continuation*	**Remission ICD-10-CM code(s)** *Continuation*
	Myeloid leukemia[†]	C9200, C922, C9210, C9212, C9220, C9222, C9230, C9232, C9240, C9242, C9250, C9252, C9260, C9262, C92A0, C92A2, C92Z0, C92Z2, C9290, C9292	Z856

International Classification of Diseases, Tenth Revision, Clinical Modification (ICD-10-CM)
[†] Note that ICD-10-CM codes with ‘in remission’ descriptor are not included

© 2024 Dicpinigaitis AJ et al. *JAMA Network Open.*

eTable 3. Finalized Adaptation of the Risk Analysis Index Parameters Into Clinically Comparable *ICD-10* Codes

RAI parameter	Code Descriptor	ICD-10-CM codes
Severe cancer‡ (5-year all-cause mortality >67%)	Malignant neoplasm of esophagus	C153, C154, C155, C158, C159
	Malignant neoplasm of liver and intrahepatic bile ducts	C220, C221, C222, C223, C224, C227, C228, C229
	Malignant neoplasm of gallbladder	C23
	Malignant neoplasm of other and unspecified parts of biliary tract	C240, C241, C248, C249
	Malignant neoplasm of pancreas	C250, C251, C252, C253, C254, C257, C258, C259
	Malignant neoplasm of trachea	C33
	Malignant neoplasm of bronchus and lung	C3400, C3401, C3402, C3410, C3411, C3412, C342, C3430, C3431, C3432, C3480, C3481, C3482, C3490, C3491, C3492
	Mesothelioma	C450, C451, C452, C457, 459
	Malignant neoplasm of brain	C710, C711, C712, C713, C714, C715, C716, C717, C718, C719
	Secondary and unspecified malignant neoplasm of lymph nodes	C770, C771, C772, C773, C774, C775, C778, C779
	Secondary malignant neoplasm of respiratory and digestive organs	C7800, C7801, C7802, C781, C782, C7830, C7839, C784, C785, C786, C787, C7880, C7889
	Secondary malignant neoplasm of other and unspecified sites	C7900, C7901, C7902, C7910, C7911, C7919, C792, C7931, C7932, C7940, C7949, C7951, C7952, C7960, C7961, C7962, C7963, C7970, C7971, C7972, C7981, C7982, C7989, C799
	Disseminated malignant neoplasm, unspecified	C800
	Secondary neuroendocrine tumors	C7B00, C7B01, C7B02, C7B03, C7B04, C7B09, C7B1, C7B8
	Acute myeloblastic leukemia; Acute myeloid leukemia with 11q23-abnormality; Acute myeloid leukemia with multilineage dysplasia†	C9200, C9202, C9260, C9262, C92A0, C92A2
	Monocytic leukemia†	C9300, C9302, C9310, C9312, C9330, C9332, C93Z0, C93Z2, C9390, C9392
Unintentional weight loss	Abnormal weight loss	R634
	Underweight	R636
	Cachexia	R64
	Adult failure to thrive	R627
	Sarcopenia	M6284
	Muscle wasting and atrophy, not elsewhere classified, unspecified state	M6250

© 2024 Dicpinigaitis AJ et al. *JAMA Network Open.*

**RAI parameter *Continued***	**Code Descriptor *Continued***	**ICD-10-CM codes *Continued***
Poor appetite	Anorexia/loss of appetite	R630
	Other feeding difficulties	R6339
	Symptoms/signs concerning for food and fluid intake	R638
	Feeding difficulties unspecified	R6330
Renal failure	Chronic kidney disease, stage 4 (severe); Chronic kidney disease, stage 5; End stage renal disease	N184, N185, N186
	Hypertensive chronic kidney disease with stage 5 chronic kidney disease or end stage renal disease	I120
	Hypertensive heart and chronic kidney disease without heart failure with stage 5 chronic kidney disease, or end stage renal disease	I1311
	Hypertensive heart and chronic kidney disease with heart failure and with stage 5 chronic kidney disease, or end stage renal disease	I132
	Encounter for care involving renal dialysis	Z4901, Z4902, Z4931, Z4932
	Dependence on renal dialysis	Z992
	Patient's noncompliance with renal dialysis	Z9115
Congestive heart failure	Heart Failure	I501, I5020, I5021, I5022, I5023, I5030, I5031, I5032, I5032, I5033, I5040, I5041, I5042, I5043, I50810, I50811, I50812, I50813, I50814, I5082, I5083, I5084, I5089, I509
	Rheumatic heart failure	I0981
	Hypertensive heart disease with heart failure	I110
	Hypertensive heart and chronic kidney disease with heart failure and stage 1 through stage 4 chronic kidney disease, or unspecified chronic kidney disease	I130
Shortness of breath	Dyspnea	R0600, R0601, R0602, R0603, R0609
	Dependence on supplemental oxygen	Z9981
	Chronic respiratory failure	J9610, J9611, J9612
**Functional dependence**		
Partial dependence	Hemiplegia and hemiparesis	G8100, G8101, G8102, G8103, G8104, G8110, G8111, G8112, G8113, G8114, G8190, G8191, G8192, G8193, G8194
	Paraplegia complete (chronic)	G8221
	Chronic paraplegia, incomplete	G8222
	Monoplegia of lower limb (not acute)	G8310, G8311, G8312, G8313, G8314
	Monoplegia of upper limb (not acute)	G8320, G8321, G8322, G8323, G8324
	Baseline need for assistance with personal care	Z741, Z742

© 2024 Dicpinigaitis AJ et al. *JAMA Network Open.*

	Code Descriptor *Continued*	ICD-10-CM codes *Continued*
	Limitation of activities due to disability	Z736
	Prior history of falling	Z9181
	Ataxic gait	R260
	Paralytic gait	R261
	Chronic difficulty in walking, not elsewhere classified	R262
	Unsteadiness on feet	R2681
	Unspecified abnormalities of gait and mobility	R269
	Age-related physical debility	R54
	Weakness	R531
	Other malaise and fatigue	R5381, R5382, R5383
	Neoplastic (malignant) related fatigue	R530
	Muscle weakness (generalized)	M6281
	Immobility syndrome (paraplegic)	M623
	Pressure ulcer	L89000, L89001, L89002, L89003, L89004, L89006, L89009, L89010, L89011, L89012, L89013, L89014, L89016, L89019, L89020, L89021, L89022, L89023, L89024, L89026, L89029, L89100, L89101, L89102, L89103, L89104, L89106, L89109, L89110, L89111, L89112, L89113, L89114, L89116, L89119, L89120, L89121, L89122, L89123, L89124, L89126, L89129, L89130, L89131, L89132, L89133, L89134, L89136, L89139, L89140, L89141, L89142, L89143, L89144, L89146, L89149, L89150, L89151, L89152, L89153, L89154, L89156, L89159, L89200, L89201, L89202, L89203, L89204, L89206, L89209, L89210, L89211, L89212, L89213, L89214, L89216, L89219, L89220, L89221, L89222, L89223, L89224, L89226, L89229, L89300, L89301, L89302, L89303, L89304, L89306, L89309, L89310, L89311, L89312, L89313, L89314, L89316, L89319, L89320, L89321, L89322, L89323, L89324, L89326, L89329, L8940, L8941, L8942, L8943, L8944, L8945, L8946, L89500, L89501, L89502, L89503, L89504, L89506, L89509, L89510, L89511, L89512, L89513, L89514, L89516, L89519, L89520, L89521, L89522, L89523, L89524, L89526, L89529, L89600, L89601, L89602, L89603, L89604, L89606, L89609, L89610, L89611, L89612, L89613, L89614, L89616, L89619, L89620, L89621, L89622, L89623, L89624, L89626, L89629, L89810, L89811, L89812, L89813, L89814, L89816, L89819, L89890, L89891, L89892, L89893, L89894, L89896, L89899, L8990, L8991, L8992, L8993, L8994, L8995, L8996

© 2024 Dicpinigaitis AJ et al. *JAMA Network Open.*

**RAI parameter** *Continuation*	**Code Descriptor** *Continuation*	**ICD-10-CM codes** *Continuation*
Total dependence	Reduced mobility (Bed confinement status/bedridden/chair-ridden/ Other reduced mobility)	Z7401, Z7409
	Need for continuous supervision	Z743
	Dependence on wheelchair	Z993
	Functional quadriplegia	R532
	Dependence on other enabling machines and devices	Z9989
	Chronic quadriplegia	G8250, G8251, G8252, G8253, G8254
Cognitive decline	Vascular dementia, unspecified severity (without / with behavioral disturbance)	F0150, F01511, F01518
	Dementia in other diseases classified elsewhere, unspecified severity (without / with behavioral disturbance)	F0280, F02811, F02818
	Unspecified dementia, unspecified severity (without / with behavioral disturbance)	F0390, F03911, F03918
	Amnestic disorder due to known physiological condition	F04
	Alzheimer disease	G300, G301, G308, G309
	Other degenerative diseases of the nervous system, not classified elsewhere (senile degeneration of the brain)	G3101, G3109, G311, G312, G3181, G3182, G3183, G3184, G3185, G3189, G319
	Age-related cognitive decline	R4181
	Alcohol-induced persisting amnestic disorder	F1096
	Alcohol dependence with alcohol-induced persisting dementia	F1027, F1097
	Sedative, hypnotic or anxiolytic dependence with sedative, hypnotic or anxiolytic-induced persisting dementia	F1327, F1397
	Inhalant use, unspecified with inhalant-induced psychotic disorder with inhalant-induced persisting dementia	F1897
	Other psychoactive substance related disorders with psychoactive substance-induced persisting dementia	F1917, F1927, F1997

International Classification of Diseases, Tenth Revision, Clinical Modification (ICD-10-CM)
Risk Analysis Index (RAI)
† Note that ICD-10-CM codes with ‘in remission’ descriptor are not included
‡ Corresponding remission ICD-10-CM Code(s) are used to limit to active cancer diagnoses

© 2024 Dicpinigaitis AJ et al. *JAMA Network Open.*

eTable 4. Final RAI-ICD Thresholds and Measures of Diagnostic Accuracy for Inpatient Mortality as Calibrated in the Validation National Inpatient Sample Population (2020)

RAI integer	Population (N)	Cumulative population (N)	Frail (%)[†]	Predicted mortality (%)	Observed Mortality (%)	Accuracy (%)	Sensitivity (%)	Specificity (%)	PPV (%)	NPV (%)	F1 score	MCC
0	65335	65335	99.19	0.31	0.23	0.00	0.00	0.00	0.00	0.00	-	-1.00
1	55	65390	99.19	0.34	0.00	0.82	99.93	0.82	0.00	100.00	0.00	0.00
2	340700	406090	94.99	0.37	0.11	0.94	99.93	0.82	0.11	99.99	0.00	0.00
3	18770	424860	94.76	0.40	1.47	6.51	99.74	5.13	1.54	99.92	0.03	0.03
4	582725	1007585	87.58	0.43	0.08	5.44	99.60	5.36	0.09	99.99	0.00	0.01
5	51290	1058875	86.95	0.46	1.47	14.00	99.36	12.72	1.67	99.92	0.03	0.04
6	696540	1755415	78.36	0.50	0.10	13.44	98.98	13.36	0.11	99.99	0.00	0.01
7	63810	1819225	77.58	0.54	1.36	23.19	98.65	22.16	1.71	99.92	0.03	0.06
8	3010	1822235	77.54	0.58	3.65	25.70	98.22	22.95	4.61	99.71	0.09	0.10
9	527220	2349455	71.04	0.63	0.36	23.26	98.17	22.99	0.46	99.97	0.01	0.03
10	3185	2352640	71.00	0.68	4.08	32.39	97.22	29.63	5.55	99.60	0.11	0.12
11	207530	2560170	68.44	0.74	0.46	29.98	97.16	29.67	0.64	99.96	0.01	0.04
12	96100	2656270	67.26	0.80	1.70	33.37	96.68	32.28	2.40	99.82	0.05	0.08
13	158195	2814465	65.31	0.86	0.72	33.92	95.87	33.47	1.03	99.91	0.02	0.05
14	111495	2925960	63.93	0.93	1.57	36.40	95.31	35.46	2.31	99.79	0.05	0.08
15	183890	3109850	61.67	1.01	0.98	37.41	94.44	36.84	1.46	99.85	0.03	0.06
16	143715	3253565	59.90	1.09	1.56	39.99	93.55	39.15	2.38	99.74	0.05	0.08
17	18360	3271925	59.67	1.17	3.27	42.62	92.44	40.93	5.02	99.38	0.10	0.12
18	407395	3679320	54.65	1.27	1.32	41.83	92.14	41.16	2.05	99.75	0.04	0.08
19	27795	3707115	54.31	1.37	3.99	47.97	89.47	46.24	6.47	99.06	0.12	0.14
20	251445	3958560	51.21	1.48	1.21	47.09	88.92	46.58	2.00	99.71	0.04	0.08
21	304460	4263020	47.45	1.60	1.98	1.73	87.41	0.00	1.74	0.00	0.03	-0.35
22	279970	4542990	44.00	1.72	1.23	53.87	84.42	53.49	2.21	99.64	0.04	0.08
23	326220	4869210	39.98	1.86	1.88	57.47	82.71	56.99	3.56	99.42	0.07	0.11
24	69440	4938650	39.12	2.01	4.43	61.86	79.66	61.03	8.65	98.48	0.16	0.17
25	586550	5525200	31.89	2.17	1.72	62.15	78.14	61.87	3.46	99.39	0.07	0.11
26	74960	5600160	30.97	2.34	4.98	69.36	73.15	69.16	11.06	98.00	0.19	0.20
27[‡]	211455	5811615	28.36	2.53	1.98	70.08	71.29	70.06	4.60	99.18	0.09	0.12
28	379895	6191510	23.68	2.73	2.85	72.58	69.21	72.68	6.91	98.77	0.13	0.15
29	137775	6329285	21.98	2.94	2.68	76.98	63.85	77.34	7.20	98.73	0.13	0.16
30	238525	6567810	19.04	3.17	2.95	78.54	62.02	79.04	8.24	98.56	0.15	0.17
31	169335	6737145	16.96	3.42	4.75	80.85	58.54	81.96	13.92	97.54	0.22	0.22
32	154270	6891415	15.05	3.69	3.74	82.90	54.55	84.00	11.69	97.94	0.19	0.19
33	89075	6980490	13.96	3.98	6.23	83.75	51.69	85.88	19.55	96.40	0.28	0.24
34	134190	7114680	12.30	4.29	5.41	86.20	45.35	88.54	18.46	96.59	0.26	0.23
35	86100	7200780	11.24	4.62	6.44	84.49	48.94	86.94	20.50	96.11	0.29	0.24
36[§]	36260	7237040	10.79	4.98	8.29	85.67	42.60	89.56	26.93	94.53	0.33	0.26
37	87575	7324615	9.71	5.36	7.45	86.34	41.11	89.98	24.81	95.00	0.31	0.25
38	56100	7380715	9.02	5.77	7.50	87.02	37.87	91.00	25.44	94.76	0.30	0.24
39	30725	7411440	8.64	6.21	7.83	87.29	35.79	91.66	26.71	94.38	0.31	0.24
40	60910	7472350	7.89	6.68	7.93	87.46	34.60	92.02	27.18	94.23	0.30	0.24

© 2024 Dicpinigaitis AJ et al. *JAMA Network Open.*

41	37595	7509945	7.43	7.18	8.70	87.46	32.20	92.73	29.66	93.49	0.31	0.24
**RAI integer *Con't***	**Population (N) *Con't***	**Cumulative population (N) *Con't***	**Frail (%)[†] *Con't***	**Predicted mortality (%)*Con't***	**Observed Mortality (%) *Con't***	**Accuracy (%) *Con't***	**Sensitivity (%) *Con't***	**Specificity (%) *Con't***	**PPV (%) *Con't***	**NPV (%) *Con't***	**F1 score *Con't***	**MCC *Con't***
42	27545	7537490	7.09	7.72	9.33	87.32	30.58	93.16	31.51	92.88	0.31	0.24
43	50520	7588010	6.47	8.30	8.00	88.34	29.31	93.48	28.08	93.83	0.29	0.22
44	73065	7661075	5.57	8.91	8.12	88.65	27.31	94.06	28.89	93.61	0.28	0.22
45	106140	7767215	4.26	9.57	7.41	89.68	24.37	94.91	27.72	94.00	0.26	0.20
46[¶]	35220	7802435	3.83	10.27	12.68	86.56	20.46	96.15	43.58	89.28	0.28	0.23
47	65930	7868365	3.01	11.01	9.06	89.45	18.25	96.54	34.47	92.22	0.24	0.20
48	109240	7977605	1.67	11.80	7.89	90.83	15.29	97.30	32.66	93.06	0.21	0.18
49	23305	8000910	1.38	12.63	13.54	86.72	11.02	98.57	54.72	87.62	0.18	0.20
50	19990	8020900	1.13	13.52	14.93	85.48	9.45	98.83	58.59	86.15	0.16	0.19
51	19625	8040525	0.89	14.46	13.91	86.37	7.97	99.04	57.36	86.95	0.14	0.18
52	10450	8050975	0.76	15.46	15.55	84.85	6.62	99.26	62.10	85.23	0.12	0.17
53	5060	8056035	0.70	16.50	15.81	84.58	5.81	99.37	63.32	84.89	0.11	0.16
54	9600	8065635	0.58	17.61	16.56	83.85	5.42	99.42	65.02	84.12	0.10	0.15
55	11235	8076870	0.44	18.77	15.89	84.45	4.63	99.52	64.68	84.67	0.09	0.14
56	2405	8079275	0.41	19.99	20.58	79.90	3.75	99.64	73.05	79.98	0.07	0.13
57	8420	8087695	0.31	21.27	18.94	81.45	3.50	99.67	71.01	81.55	0.07	0.13
58	10915	8098610	0.17	22.61	17.96	82.33	2.71	99.75	70.54	82.41	0.05	0.11
59	1905	8100515	0.15	24.00	26.25	74.11	1.74	99.87	82.14	74.07	0.03	0.09
60	3190	8103705	0.11	25.46	26.33	73.97	1.49	99.88	82.03	73.94	0.03	0.09
61	2100	8105805	0.09	26.97	23.10	77.09	1.07	99.91	78.75	77.08	0.02	0.07
62	515	8106320	0.08	28.54	21.36	78.77	0.83	99.93	77.27	78.77	0.02	0.07
63	945	8107265	0.07	30.16	26.98	73.18	0.78	99.94	82.40	73.16	0.02	0.06
64	790	8108055	0.06	31.83	20.89	79.21	0.65	99.95	76.55	79.21	0.01	0.06
65	1060	8109115	0.04	33.54	20.75	79.33	0.57	99.96	76.92	79.33	0.01	0.05
66	335	8109450	0.04	35.31	35.82	64.32	0.46	99.97	88.27	64.28	0.01	0.05
67	830	8110280	0.03	37.11	24.70	75.38	0.40	99.97	80.71	75.37	0.01	0.05
68	1385	8111665	0.01	38.95	23.83	76.23	0.30	99.98	79.92	76.22	0.01	0.04
69	310	8111975	0.01	40.83	27.42	72.61	0.14	99.99	83.41	72.61	0.00	0.03
70	305	8112280	0.01	42.73	27.87	72.15	0.09	99.99	83.10	72.15	0.00	0.02
71	285	8112565	0.00	44.65	21.05	78.95	0.05	100.00	75.07	78.96	0.00	0.02
72	65	8112630	0.00	46.59	15.38	84.62	0.02	100.00	69.62	84.62	0.00	0.01
73	75	8112705	0.00	48.53	26.67	73.34	0.02	100.00	85.50	73.34	0.00	0.01
74[#]	40	8112745	0.00	50.49	25.00	75.00	0.01	100.00	86.71	75.00	0.00	0.01

Risk Analysis Index (RAI)
Negative predictive value (NPV)
Positive predictive value (PPV)
Matthews Correlation Coefficient (MCC)
[†] Frail (%) indicates the proportion of the sample identified as "frail" if the cutoff is set for the row's integer value of RAI (e.g., at RAI = 22 the proportion of the population with RAI ≥ 22 is 44%)
[‡] Normal cutoff
[§] Frail cutoff
[¶] Very frail cutoff
[#] The theoretical range of RAI is 0-81, but in this sample, no values above 74 were observed
Rows with no shading indicate "robust" RAI scores; deepening shades of green indicate increasing strata of frailty (e.g., normal, frail, and very frail). Following methods established previously for the RAI-A and RAI-C,[10] the RAI-ICD integer of 36 defined the frailty cutoff point since predicted mortality at this level (4.98%) is approximately double that of the mortality observed within the analyzed

© 2024 Dicpinigaitis AJ et al. *JAMA Network Open.*

validation population (2.5%). This cutoff also correlates with maximal F1 score (0.33) and MCC (0.26) values representing an optimal balance between sensitivity and specificity, accuracy, and precision. The cut point of 27 was selected as cutoff for normal strata because its predicted mortality approximates the mortality in the studied population (2.5%). Patients with RAI-ICD below 27 are considered robust because their predicted mortality is less than the 2.5% mean mortality in the studied population. The cutoff of 46 was selected to delineate the severely frail since predicted mortality is approximately four times of the studied population mortality (2.5% vs 10.3%). Therefore, suggested RAI-ICD groups are robust (<27), normal (27-35), frail (36-45), and very frail (>45)

© 2024 Dicpinigaitis AJ et al. *JAMA Network Open.*

eFigure1. Survey-Weighted and Estimated Populations in National Inpatient Sample (NIS) for the (A) Derivation and (B) Validation Cohorts

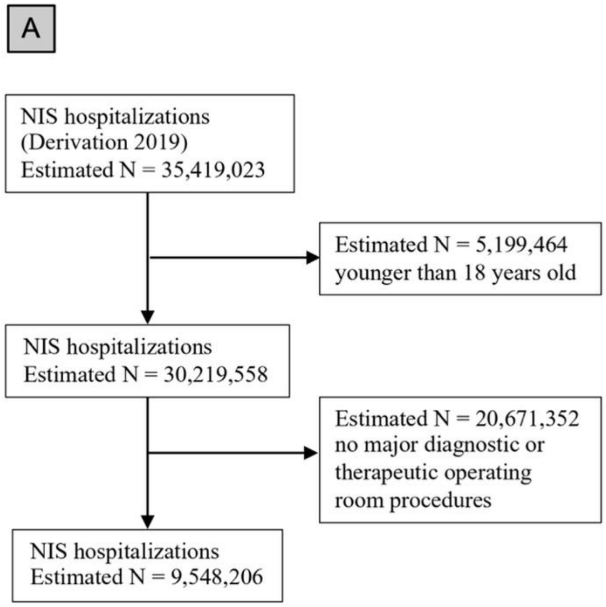

Numbers (N) are given as population estimates generated from the survey weight as all since analysis was performed on survey-weighted data. Major diagnostic or therapeutic operating room procedures were identified based on NIS indicator for major operating room ICD-10 Procedural Coding System procedure (PCLASS_ORPROC).

© 2024 Dicpinigaitis AJ et al. *JAMA Network Open.*

eTable 5. Data Missingness in Operative National Impatient Populations Among Adult Hospitalizations

Variable name	Proportion missing
Derivation population (National Inpatient Population 2019)	
Sex	0.02 (0.01-0.02)
Age	0.00 (0.00-0.00)
Race	2.53 (2.13-3.00)
In-hospital mortality†	0.02 (0.01-0.02)
Elective admission	0.15 (0.11-0.20)
Length of hospital stay	0.00 (0.00-0.00)
Total charges	0.45 (0.26-0.77)
Validation population (National Inpatient Population 2020)	
Sex	0.02 (0.01-0.02)
Age	0.00 (0.00-0.00)
Race	2.69 (2.28-3.17)
In-hospital mortality†	0.02 (0.01-0.02)
Elective admission	0.15 (0.11-0.19)
Length of hospital stay	0.00 (0.00-0.00)
Total charges	0.84 (0.48-1.47)

Variables are presented as proportion (95% confidence interval). All data are survey weighted.
† In-hospital mortality missingness is calculated based on discharge disposition NIS variable prior to recategorization of our primary outcome with ICD-10-CM initiating “transition to comfort care” (Z51.5) code

Hospitalizations with missing data were not omitted from the analysis; however, these missing data were inherently unavailable for scoring of the RAI-ICD. Therefore, missing data are likely to skew the RAI-ICD towards the null. However, those hospitalizations without age or sex data or data for the primary outcome were excluded from the modeling.

The included UPMC data used in sensitivity analysis does not have missingness for sex or age.

eTable 6. Logistic Regression for In-Hospital Mortality Weighting Risk Analysis Index Parameters in the National Inpatient Sample Derivation Population (2019)

Risk Analysis Index Parameter	β	95% CI	P-value
Age	0.033	0.032-0.034	< 0.001
Age*cancer[†]	-0.030	-0.033- -0.028	< 0.001
Cancer[†]	3.675	3.498-3.852	< 0.001
Male sex	0.229	0.207-0.251	< 0.001
Weight loss	0.158	0.151-0.164	< 0.001
Poor appetite	0.063	0.042-0.085	< 0.001
Renal failure	0.181	0.172-0.190	< 0.001
Congestive heart failure	0.252	0.245-0.259	< 0.001
Shortness of breath	0.115	0.089-0.142	< 0.001
Functional status	0.739	0.713-0.764	< 0.001
Cognitive decline	0.683	0.638-0.728	< 0.001
Functional status*cognitive decline	-0.312	-0.366- -0.257	< 0.001

Confidence interval (CI)
* Denotes statistical interaction term
[†] Cancer variable is based on severe categorization defined by 5-year all-cause mortality >67%

© 2024 Dicpinigaitis AJ et al. *JAMA Network Open.*

eResults.

Following integerization the RAI-ICD model performance was evaluated for each cancer categorization: severe, moderate, and mild. The performance of models on Decision Curve Analysis was equivocal (p 5). Optimal performance with the highest C-statistics (p 17) and the best balance between predicted mortality, F1 score, and Mathews Correlation Coefficient (MCC) performance was achieved with severe cancer categorization. The proportion of observed and predicted mortality 95% confidence interval overlap across cancer groups was as following: severe – 37.8%, moderate – 36.0%, mild – 31.1%. Based on these results, the severe cancer categorization and corresponding ICD-10-CM codes were selected as the final cancer parameter definition for the final RAI-ICD.

© 2024 Dicpinigaitis AJ et al. *JAMA Network Open.*

eFigure2. Decision Curve Analysis for RAI-ICD for Alterative Cancer Parameter Definitions

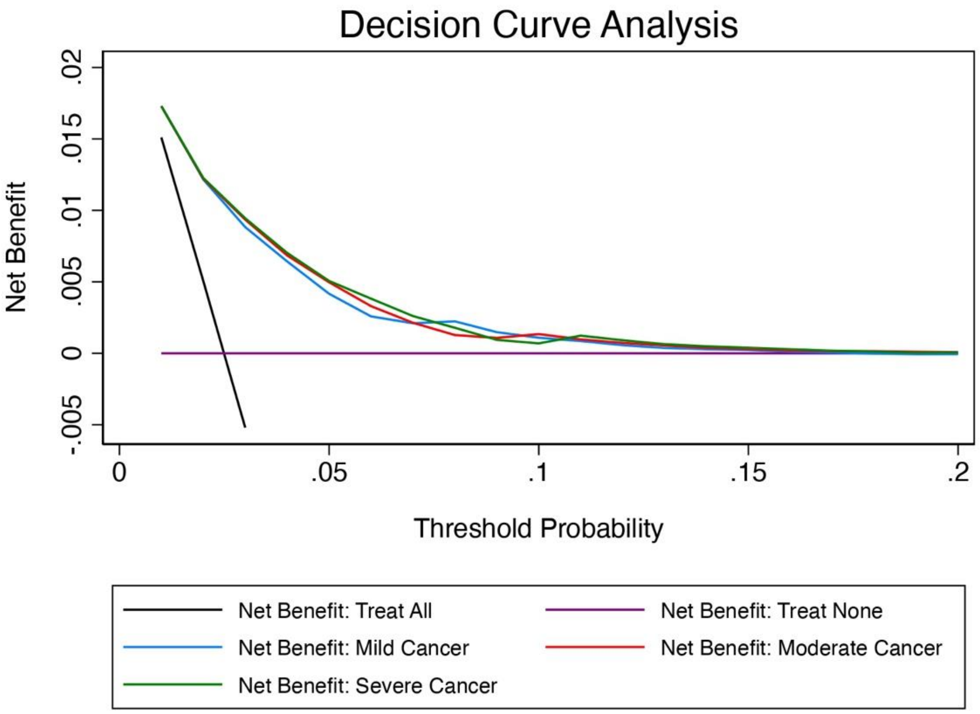

Decision curve analysis comparing RAI-ICD performance based on International Classification of Diseases, Tenth Revision, Clinical Modification codes for altering cancer definitions based upon the estimated 5-year – mild cancer (blue), moderate cancer (red line), severe cancer (green line) against treat all (black line) and treat none (purple line).

© 2024 Dicpinigaitis AJ et al. *JAMA Network Open.*

eTable 7. Model Performance Based on *ICD-10-CM* Codes for Each Cancer Categorization in National Inpatient Sample Derivation Population (2019)

**Cancer categorization**	C-statistic<br>(95% Confidence Interval)	AIC†	BIC†	Overlap
Severe	0.784 (0.782-0.786)	346708.8	346733.7	37.8%,
Moderate	0.784 (0.782-0.786)	347057.9	347082.8	36.0%,
Mild	0.779 (0.777-0.782)	348541.7	348566.6	31.1%.

International Classification of Diseases, Tenth Revision, Clinical Modification (ICD-10-CM)
Akaike's information criterion (AIC)
Bayesian information criterion (BIC)
† As limited by available data processing AIC and BIC were calculated on an unweighted derivation sample

© 2024 Dicpinigaitis AJ et al. *JAMA Network Open.*

eTable 8. Baseline Characteristics of Sensitivity Cohorts Including Operative and Non-Operative Hospitalizations

Variable	UPMC Sample (N=1,316,544)	National Inpatient Sample Sensitivity Population (Estimated N=27,668,666)
**Demographics**		
Age (years)	52.4 (0.02)	58.0 (0.1)
Sex		
Male	43.5 (43.4-43.6)	43.8 (43.6-44.1)
Female	56.5 (56.4-56.6)	56.2 (55.9-56.4)
Race		
Asian	1.1 (1.1-1.2)	2.8 (2.5-3.0)
Black	17.2 (17.2-17.3)	15.4 (14.8-16.1)
White	78.0 (77.9-78.1)	64.0 (63.0-65.0)
Other†	1.9 (1.8-1.9)	15.3 (14.5-16.1)
Missing‡	1.8 (1.7-1.8)	2.5 (2.2-3.0)
**Risk Analysis Index Parameters**		
Cancer§	2.6 (2.5-2.6)	5.1 (4.9-5.3)
Weight loss	1.7 (1.7-1.7)	2.9 (2.8-2.9)
Poor appetite	0.2 (0.2-0.3)	0.4 (0.4-0.5)
Renal failure	2.4 (2.3-2.4)	6.1 (6.0-6.2)
Congestive heart failure	8.4 (8.4-8.5)	18.7 (18.5-19.0)
Shortness of breath	3.3 (3.3-3.4)	4.6 (4.5-4.7)
Cognitive decline	2.7 (2.6-2.7)	6.6 (6.5-6.7)
Functional status		
Independent	94.2 (94.2-94.3)	89.3 (89.1-89.5)
Partially dependent	4.7 (4.7-4.8)	8.6 (8.5-8.8)
Totally dependent	1.1 (1.0-1.1)	2.1 (2.0-2.1)
**Risk Analysis Index score**		
RAI-ICD integer	18.9 (0.01)	25.3 (0.1)
RAI-ICD category		
Robust	73.7 (73.6-73.8)	59.5 (59.1-59.9)
Normal	17.0 (16.9-17.0)	22.3 (22.1-22.5)
Frail	6.2 (6.2-6.3)	11.4 (11.3-11.5)
Very Frail	3.1 (3.1-3.1)	6.8 (6.7-6.9)

Continuous variables are presented as means (standard error) and categorical as proportion (95% confidence interval). All data are survey weighted.

† Race and ethnicity are categorized within National Inpatient Sample in one single data element. Other race includes Hispanic, Native American, and other

‡ Reported if >0.1% of variables are missing

§ Cancer variable is defined based on International Classification of Diseases, Tenth Revision, Clinical codes for severe cancer categorization (p 12)

© 2024 Dicpinigaitis AJ et al. *JAMA Network Open.*

eFigure 3. Observed and Predicted Mortality by the Integerized RAI-ICD Score for Sensitivity Analysis

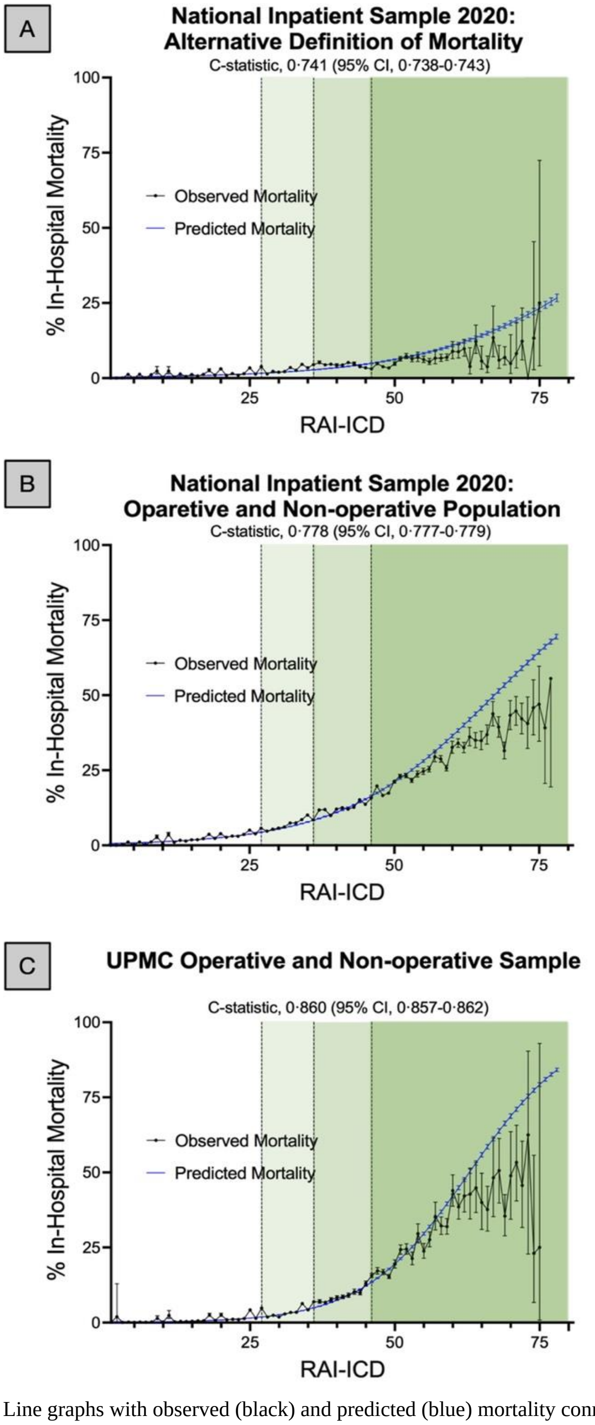

Line graphs with observed (black) and predicted (blue) mortality connecting survey-weighted, population-based point estimates (dot) and associated 95% confidence intervals (error bars) across the integerized RAI-ICD. The dashed horizontal lines separate frailty categories with the white background (RAI-ICD, <27) indicating robust, lightest green (RAI-ICD, 27-35) normal, mid-shade green (RAI-ICD, 36-45) frail, and darkest green (RAI-ICD, >45) very frail. Data are demonstrated for the sensitivity analysis National Inpatient Sample, 2020 with alterative definition of in-hospital mortality (N=8,113,950; Panel A); National Inpatient Sample, 2020 with and without major diagnostic or therapeutic operating room procedures (N=27,668,666; Panel B) populations; UPMC, 2021-2022 sample (N=1,316,544; Panel C).

© 2024 Dicpinigaitis AJ et al. *JAMA Network Open.*

eTable 9. Spearman Correlation Between Various Frailty Tools

	VA-FI ρ (95% confidence interval)	HFRS ρ (95% confidence interval)
VA-FI	..	..
HFRS	0.669 (0.669-0.670)	..
RAI-ICD	0.701 (0.700-0.702)	0.560 (0.559-0.561)

VA Frailty Index (VA-FI)
Hospital Frailty Risk Score (HFRS)
Data provided are the Spearmen correlation coefficients (ρ) with the corresponding 95% confidence intervals.

© 2024 Dicpinigaitis AJ et al. *JAMA Network Open.*

eFigure 4. Distribution of Frailty Categorization Among UPMC Hospitalizations With an Available Outpatient Risk Analysis Index – Clinical (RAI-C) Score

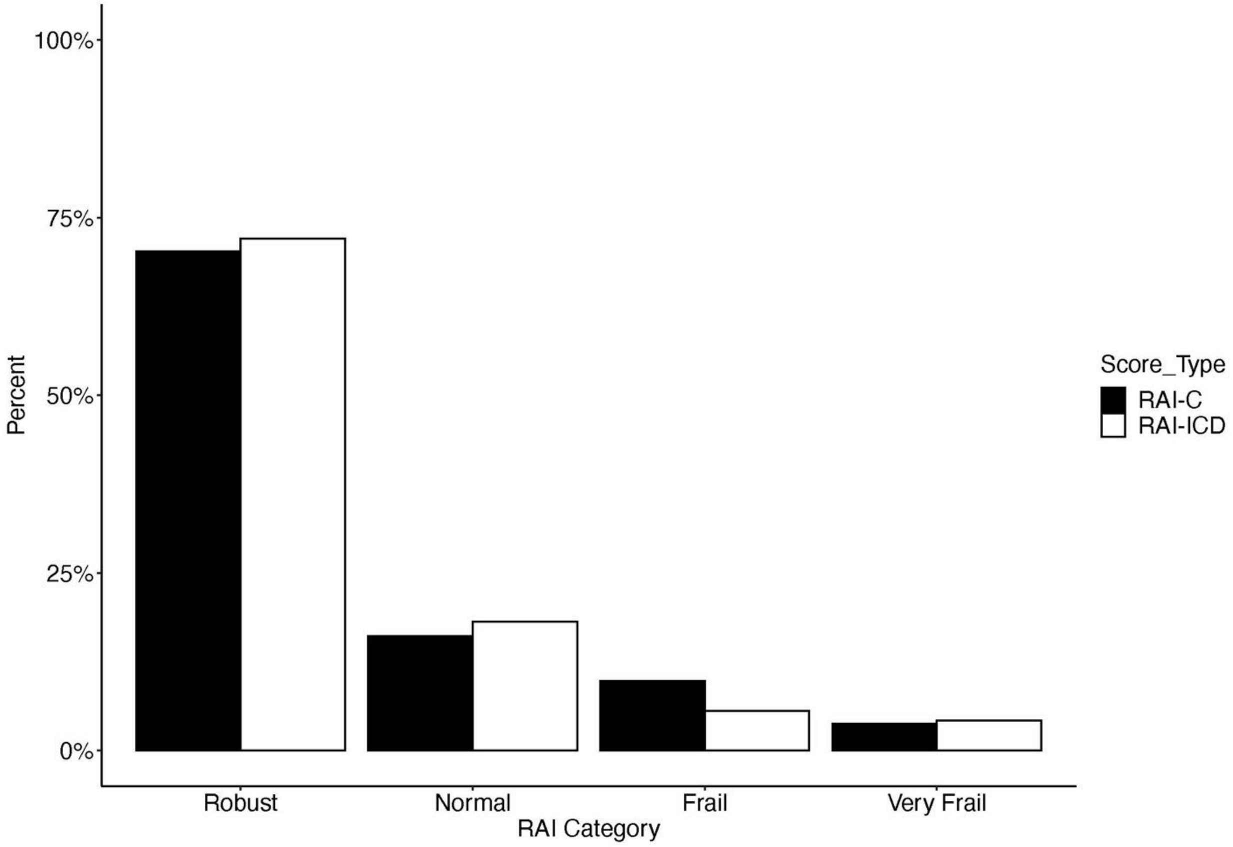

Among the subset of patients at UPMC with an RAI-C captured within 90 days of the hospitalization (N=63,860/1,316,544 [5%]), the bar graph documents the proportion of patients categorized as Robust, Normal, Frail, and Very Frail. Cut offs vary slightly within the scoring systems: RAI-C: RAI ≤ 29 Robust, 30 ≤ RAI ≤ 36 Normal, 37 ≤ RAI ≤ 44 Frail, RAI ≥ 45 Very Frail. RAI-ICD: RAI < 27 Robust, 27 ≤ RAI ≤ 35 Normal, 36 ≤ RAI ≤ 45 Frail, RAI > 45 Very Frail.

© 2024 Dicpinigaitis AJ et al. *JAMA Network Open.*

eReferences

1. Orkaby AR, Nussbaum L, Ho YL, et al. The Burden of Frailty Among U.S. Veterans and Its Association With Mortality, 2002-2012. *J Gerontol A Biol Sci Med Sci*. 2019;74(8):1257-1264.
2. Gautam N, Bessette L, Pawar A, Levin R, Kim DH. Updating International Classification of Diseases 9th Revision to 10th Revision of a Claims-Based Frailty Index. *J Gerontol A Biol Sci Med Sci*. 2021;76(7):1316-1317.
3. The Web's Free 2023 ICD-10-CM/PCS Medical Coding Reference. https://www.icd10data.com/ (accessed Feb 8, 2023).
4. Neoplasms. https://www.icd10data.com/ICD10CM/Codes/C00-D49 (accessed Feb 8, 2023).
5. SEER*Explorer: An interactive website for SEER cancer statistics. Surveillance Research Program, National Cancer Institute. https://seer.cancer.gov/statistics-network/explorer/. (accessed Feb 10, 2023).
6. American Cancer Society. Cancer Statistics Center. http://cancerstatisticscenter.cancer.org (accessed Feb 10, 2023).
7. Akaike H. Information Theory and an Extension of the Maximum Likelihood Principle. In: Parzen E, Tanabe K, Kitagawa G, editors. Selected Papers of Hirotugu Akaike. New York, NY: Springer New York; 1998. p. 199-213.
8. Schwarz G. Estimating the Dimension of a Model. *The Annals of Statistics*. 1978;6(4):461-4.
9. van Walraven C, McAlister FA, Bakal JA, Hawken S, Donzé J. External validation of the Hospital-patient One-year Mortality Risk (HOMR) model for predicting death within 1 year after hospital admission. *CMAJ*. 2015;187(10):725-733.
10. Arya S, Varley P, Youk A, et al. Recalibration and External Validation of the Risk Analysis Index: A Surgical Frailty Assessment Tool. *Ann Surg*. 2020;272(6):996-1005.

© 2024 Dicpinigaitis AJ et al. *JAMA Network Open.*
