# Supplementary material for: Adaptation of the Risk Analysis Index for Frailty Assessment Using Diagnostic Codes
Source: JAMA Netw Open. 2024 May 24;7(5):e2413166. doi: 10.1001/jamanetworkopen.2024.13166 (PMC11127118; doi:10.1001/jamanetworkopen.2024.13166)
Supplement: Supplement 2. — Data Sharing Statement [file jamanetwopen-e2413166-s002.pdf]

Data Sharing Statement

Dicpinigaitis. Adaptation of the Risk Analysis Index for Frailty Assessment Using Diagnostic Codes. *JAMA Netw Open*. Published May 24, 2024.
doi:10.1001/jamanetworkopen.2024.13166

Data
Data available: No

Additional Information
Explanation for why data not available: The data are publically available through the HCUP. We have made our analysis and the coding publicly available (GitHub) for the recapitulation of our results.
